# Effectiveness assessment of a home-based exercise intervention in mitigating HTLV-1 associated disabilities: A validation study

**DOI:** 10.1371/journal.pone.0302542

**Published:** 2024-05-14

**Authors:** Izabela Mendonça de Assis, Bianca Callegari, Maísa Silva de Sousa

**Affiliations:** 1 Tropical Medicine Center, Federal University of Pará, Belém, Pará, Brazil; 2 Health Sciences Institute, Faculty of Physiotherapy, Federal University of Pará, Belém, Pará, Brazil; Chinese Academy of Sciences, CHINA

## Abstract

To evaluate the effectiveness of a home exercise program called Home Exercise Booklet for People Living with Human T Lymphotropic Virus 1 (HTLV-1). This is a methodological study of content validation with expert judges. A questionnaire with a Likert scale was applied, containing 16 items referring to the content domain. Descriptive statistics were used to obtain the content validity index. In total, 46 judges participated, 24 physiotherapists (PG) and 22 professionals from other health areas specializing in methodological studies and HTLV-1 (EG). In the validation process, each evaluator judged the technology and scored their considerations. In the end, we obtained the following results for the Content Validity Index (CVI): PG CVI: 94.3%, GE CVI: 93.4%. Although the index was sufficient to consider the technology validated, modifications were made to the second and final version of the booklet, considering the judges’ observations and suggestions, which we consider relevant. The technology proved to be valid for use with the target audience. The development and validation of this product provides support to help prevent functional decline in people living with HTLV-1; standardize guidelines for physiotherapy professionals who monitor these issues; start a home exercise program aimed at other comorbidities; open the possibility of creating and validating home exercise programs with other comorbidities.

## Introduction

Health education technologies (HET) are a set of knowledge that enable the prevention of diseases and rehabilitation of people. They can, therefore, support health education actions, as they are instruments that seek to build knowledge and prepare people to take control and responsibility for self-care, as well as encouraging them to empower themselves, make decisions and act on the conditions and determinants of their health and quality of life [1, 2].

The Pasquali validation model is a methodology from the Theory of Psychometrics that involves the development of instruments for measuring subjective phenomena in different areas of health, with the composition of three poles or procedures: theoretical, which concerns the development of technology; empirical, referring to the discussion of the phases and techniques of how to apply the pilot technology, as well as the collection of information to be able to evaluate the psychometric properties of this technology; analytical, consisting of statistical analysis of data to obtain a valid and reliable technology [3].

For Raymundo [4], validation is an entire investigation process since, rather than exhausting itself, can continue and repeat itself countless times for the same instrument. In the context of health practice, educational technologies have been produced, but the vast majority are not validated or evaluated. Educational materials such as folders, posters, booklets, manuals, guidance notebooks or handouts are usually made available to the population before being tested and are not always (or almost never) subjected to a validation process. This is mainly due to the fact that many health professionals are not aware of this process [5].

The human T-lymphotropic virus 1 (HTLV-1) is a retrovirus in the Retroviridae family that affects human blood T lymphocytes and can lead to neurological disorders. It shows a silent, long-lasting persistence in hosts and, despite its irregular distribution, estimates suggest that at least 20 million people are infected with HTLV-1 worldwide [6–10].

About two million people live with the infection in Brazil. This number, however, is heterogeneously distributed, varying according to the geographic region [8, 11]. In general, HTLV-1 infection is associated with unfavorable socioeconomic and educational indicators, with women being the most affected population [12, 13].

The development of serious diseases has been found in association with the virus, such as tropical spastic paraparesis or HTLV-1 associated myelopathy/tropical spastic paraparesis (HAM/TSP). This neurological condition is characterized by the onset of motor disabilities and a slowly progressive and non-remitting inflammation of the spinal cord, affecting 4 to 5% of infected subjects. It causes more proximal motor weakness, spasticity of the lower limbs, pain, and bladder, intestinal, and sexual dysfunctions, and thus functional limitations such as impaired walking, going up and down stairs, hygiene, dressing, and urinary continence [14].

Faced with this situation of important motor disabilities, physical therapy has been prescribed for neurological complications associated with HTLV-1 as it contributes to improve functional status, reduce symptoms, and positively impact people’s quality of life [15–17]. Considering the importance of implementing physical exercise programs, the development of protocols that can be carried out at home is indicated as an alternative treatment and continued care in the search for a solution for existing health conditions. Healthcare providers must constitute methodologies that continuously stimulate patients and the teaching-learning process. Strategies to encourage adherence and motivation are fundamental for the success of treatment performed at home without the direct guidance of healthcare providers [18].

Therefore, it is important to evaluate the effectiveness of a home exercise intervention in mitigating disabilities associated with HTLV-1 through a validation study to contribute to the prevention of functional decline in people living with HTLV-1 and safely support the regular practice of physical exercises specific to this population in an autonomous, conscious, and responsible manner.

## Methods

This is a methodological study with emphasis on content validation that used the opinion of specialist judges and experts. Methodological research focuses on production, testing, and/or validation processes, as well as the improvement of different devices and methodological strategies [19].

This study was submitted to the Ethics Committee for Research Involving Human Beings from the Nucleus for Research in Oncology at Federal University of Pará, following the norms of Resolution 486/12 of the National Health Council regarding research in human beings, obtaining approval under opinion N°. 4.424.832 on November 27, 2020.

The HET evaluation forms and informed consent forms were coded to guarantee judges’ anonymity, explained to participants, and applied before the evaluation of the booklet. All participants were invited to participate by email. Those who responded received the consent form and, after their formal acceptance, were sent the HET evaluation forms and the educational technology in a digital document in Portable Document Format (PDF).

A literature review was carried out to produce the HET [20]. Then, after reading the final sample, themes that could contribute to the composition of the first version were selected [21]. No other product created for this purpose was found in the review.

The final version of the technology called “Home Exercise Booklet for People Living with HTLV-1” was created in the Portuguese language, with 36 pages, consisting of a cover, back cover, technical sheet, summary, presentation page and information about HTLV-1 and PDE. His illustrations portray the ethnic diversity of the Brazilian population, mainly showing women with different biotypes in their fourth decade of life (S1 File).

The proposed booklet is an instrument that offers the possibility of standardizing, guiding, and encouraging healthy lifestyle habits by exercise within the reality and possibilities of the involved population. In addition to exercises, the booklet offers guidelines to define the disease, signs, and symptoms, forms of transmission, prevention, and treatment, which are important to determine in which situations participants should seek health care, interrupt or postpone the performance of the exercises, and remedy their doubts.

The search for the selection of physical therapists and specialists in HTLV and methodological studies was in line with the objectives of this research since chosen participants should have affinity with the proposal to be validated. Therefore, they should have academic, scientific, and/or professional experience in the studied area. Physical therapy judges considered for this study were professionals with experience in care, teaching, and/or research and expert judges in the field of HTLV and methodological studies had been researchers, professors, and/or technical coordinators from different areas of work in health for at least five years.

Given this context, it was necessary for a multidisciplinary team to evaluate the construction of the technology, since the development and validation process involves scientific knowledge that aims to support research with scientific evidence. We seek to select judges from the most diverse areas and specialties aligned with the objectives of this study.

Two distinct judging committees were set up: a first group (PG) made up of physiotherapy professionals and a second (GE) made up of professionals from other health areas specializing in methodological studies and HTLV-1 (GE). In the validation process, the Pasquali Model was chosen, which requires the completion of steps ranging from the construction of the instrument, obtaining the opinion of the judges to the application of different statistical procedures [3]. The judges were contacted via an invitation letter sent by email. After acceptance, the Informed Consent Form, the HET and an evaluation form were sent. A period of 10 days was established for the groups, during which the judges read the HET and completed the form.

Sample calculation to determine the number of judges for content validation was obtained using the formula n = Za2 P(-1-P)/e2. The stipulated values were Za (confidence level) = 95%, P (proportion of judge’s agreement) = 85%, and (accepted difference from expectation) = 15%, resulting in 22 judges [22].

To assist the analysis of research judge committees, a specific evaluation form was prepared for each committee with two questionnaires in each form. Questionnaire A contained questions related to evaluators’ personal/professional characterization and questionnaire B, the 16 items related to the content of the HET, which was adapted to its electronic form version (Google Forms) to be sent by email to research judges.

To issue their opinion, judges read the HET and filled out the form with their responses, as shown as a Likert-type scale: 1. Inadequate; 2. Partially adequate; 3. Adequate; 4. Completely adequate.

The statistical tests used were: Chi-square test of contingency and adherence to verify whether the frequency with which a given event observed in the sample of judges deviated significantly or not from the frequency with which it was expected; Student’s T test, to verify that the scores of PG and GE individuals did not differ in relation to the means; Bland-Altman graph, to evaluate the CVI agreement between the two groups of judges (HIRAKATA, CAMEY, 2009). All results were considered statistically significant at a significance level of 5% (p≤0.05).

The content validity index (CVI) was used to measure the proportion or percentage of judges who agreed on certain aspects of the HET and its items, analyzing each item individually and then the instrument as a whole. The CVI score is calculated by adding judges agreements on items marked as “3” or “4” by specialists. The CVI in this study for each item analyzed was considered valid if it was greater than 78% or 0.78 [23].

The formula to evaluate each item individually is the following:

CVI = number of “Adequate” or “Completely adequate” responses/ total number of answers

The instrument used to validate the technology was composed of items, distributed in domains, according to research with a similar purpose in validating an educational booklet, with a physical exercise program to prevent falls, carried out at the University of Brasília [24]: 1. Content (16 items); 2. Language (8 items); 3. Images and Illustrations (5 items); 4. Layout and Design (14 items); 5. Physiotherapy exercises (7 items). The PG responded to domains 1, 2, 3, 4 and 5 and the EG to domains 1, 2, 3 and 4.

## Results

We invited 107 professionals (52 physical therapists and 55 professionals from other health areas specializing in methodological studies and HTLV-1), of which five refused to participate, six did not deliver the evaluation after accepting the invitation, and 50 did not respond to the invitation. Thus, 46 judges (24 physical therapists and 22 experts) evaluated the booklet by a validation form. After completing them, the judges sent them to the researcher via Google Forms, along with the appropriate suggestions for modifying the material. We descriptively analyzed data on the characterization of judge groups participating in this study according to previously established inclusion criteria.

Of the total sample of judges, most participants were women (60.9%) aged from 31 to 40 years (47.9%) with a training time of 05 to 10 years (21.7%), with higher education degrees—most often in physical therapy (52.2%), biomedicine (15.2%) and medicine (10.9%)—, with a PhD as their higher degree (37.0%), and with experience in caring for patients with HTLV (76.1%) (Table 1).

**Table 1:** General characteristics of the sample according to a group of physical therapists (PG) and a group of professionals from other health areas specializing in methodological studies and HTLV-1 (EG).

Variable	PG (n = 24)		EG (n = 22)		Total (n = 46)		p-value*
	n	%	n	%	n	%	
**Sex**							
Female	14	58.3%	14	63.6%	28	60.9%	0.9476
Male	10	41.7%	08	36.4%	18	39.1%	
**Age**							
25–30	06	25.0%	02	09.1%	08	17.4%	0.1780
31–40	13	54.2%	09	40.9%	22	47.9%	
41–50	02	08.3%	04	18.2%	06	13.0%	
> 50	03	12.5%	07	31.8%	10	21.7%	
**Higher education area**							
Biomedicine	00	00.0%	07	31.8%	07	15.2%	na
Biological Sciences	00	00.0%	03	13.6%	03	06.5%	
Nursing	00	00.0%	02	09.1%	02	04.3%	
Pharmacy	00	00.0%	03	13.6%	03	06.5%	
Physical therapy	24	100.0%	00	00.0%	24	52.2%	
Medicine	00	00.0%	05	22.7%	05	10.9%	
Occupational therapy	00	00.0%	01	04.6%	01	02.2%	
Veterinary	00	00.0%	01	04.6%	01	02.2%	
**Time since graduation (graduation)**							
< 5 years	06	25.0%	01	04.6%	07	15.2%	0.1899
Between 5 and 10 years	06	25.0%	04	18.2%	10	21.7%	
Between 10 and 15 years	03	12.5%	04	18.2%	07	15.2%	
> 15 years	09	37.5%	13	59.0%	22	47.9%	
**Highest degree**							
Specialization	06	25.0%	00	00.0%	06	13.0%	0.0741
Master’s degree	07	29.2%	06	27.3%	13	28.3%	
PhD	07	29.2%	10	45.4%	17	37.0%	
Post doctorate	04	16.6%	06	27.3%	10	21.7%	
**Experience with a patient with HTLV**							
Yes	17	70.8%	18	81.8%	35	76.1%	0.5985
No	07	29.2%	04	18.2%	11	23.9%	
**Participation in study groups**							
Yes	19	79.2%	20	90.9%	39	84.8%	0.4860
No	05	20.8%	02	09.1%	07	15.2%	
**Works at a higher education institution**							
Yes	22	91.7%	22	100.0%	44	95.7%	0.5088
No	02	08.3%	00	00.0%	02	04.3%	

PG = Physiotherapists group; EG = professionals from other health areas specializing in methodological studies and HTLV-1 Group; na = Not applicable.

*Contingency chi-square test (row × column).

As for participation in research groups, most volunteers act as participating members (84.8%) and work at a higher education institution (95.7%).

Our frequency distribution analysis found that no characteristic showed statistical differences in both groups, evincing homogeneity between the two samples.

Before evaluating the form, we instructed the judges to write their suggestions for the material below each domain of the evaluation instrument.

**Fig 1:**
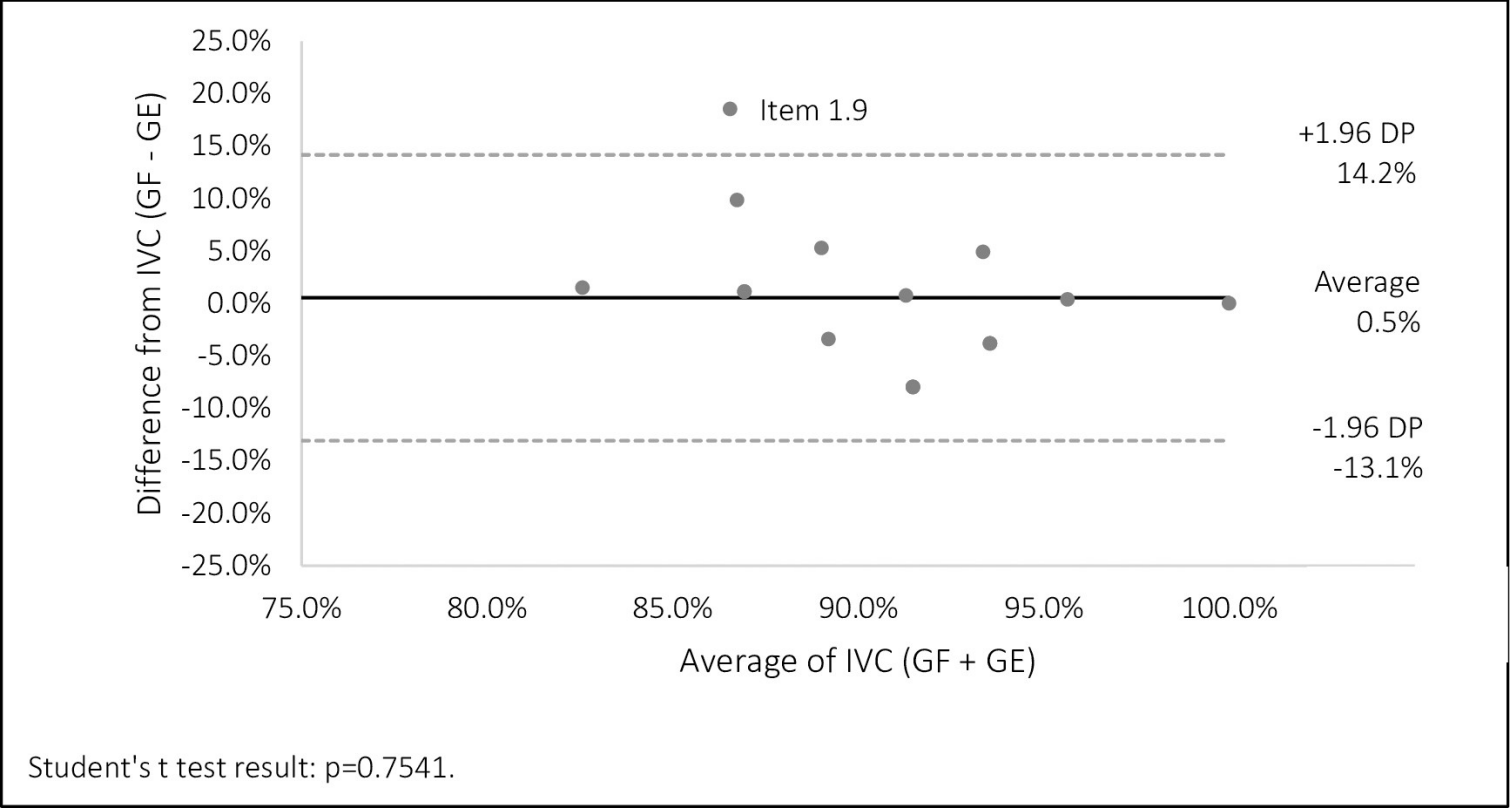
Analysis using the Bland-Altman plot.

We describe the results of our CVI calculations below, in which we show the CVI of the items of each domain of the assessment instrument. After evaluation by the judges, the booklet obtained an overall CVI ranging from 93.4% to 94.3%, thus being considered validated according to the cutoff point adopted. In general terms, we found that, in addition to the CVI of each domain reaching the validation index, domain items showed agreement since the average of the observed differences of the CVI between PG and EG failed to significantly differ from zero, remaining within the confidence interval (Fig 1).

Next, we will detail the CVI results of each domain in each group of research judges.

Regarding the validation process of the booklet for Content, judges answered the 16 items of the referred domain in the evaluation instrument. Only one item in the EG obtained a CVI below that considered appropriated (item 1.9, with 77.3%). Judges classified the other items as suitable or adequate, giving a CVI of up to 100% in both groups (Tables 2 and 3).

**Table 2:** Absolute frequencies of responses related to content evaluation and CVI in the physical therapist group.

Content of the booklet	PG (n = 24)		CVI	p-value
	Answer 1–2	Answer 3–4		
Item 1.1	03	21	87.5%	0.0005*
Item 1.2	01	23	95.8%	< 0.0001*
Item 1.3	02	22	91.7%	0.0001*
Item 1.4	02	22	91.7%	0.0001*
Item 1.5	03	21	87.5%	0.0005*
Item 1.6	02	22	91.7%	0.0001*
Item 1.7	03	21	87.5%	0.0005*
Item 1.8	02	22	91.7%	0.0001*
Item 1.9	01	23	95.8%	< 0.0001*
Item 1.10	02	22	91.7%	0.0001*
Item 1.11	03	21	87.5%	0.0005*
Item 1.12	04	20	83.3%	0.0022*
Item 1.13	03	21	87.5%	0.0005*
Item 1.14	03	21	87.5%	0.0005*
Item 1.15	00	24	100.0%	< 0.0001*
Item 1.16	01	23	95.8%	< 0.0001*

PG = Physical therapist group.

*Significant result for the Chi-square Test of adherence.

**Table 3:** Absolute frequencies of responses related to content evaluation and CVI in the HTLV-1 professionals from other health areas specializing in methodological studies and HTLV-1 group.

Content of the booklet	EG (n = 22)		CVI	p-value
	Answer 1–2	Answer 3–4		
Item 1.1	03	19	86.4%	0.0014*
Item 1.2	02	20	90.9%	0.0003*
Item 1.3	01	21	95.5%	< 0.0001*
Item 1.4	02	20	90.9%	0.0003*
Item 1.5	03	19	86.4%	0.0014*
Item 1.6	04	18	81.8%	0.0056*
Item 1.7	01	21	95.5%	< 0.0001*
Item 1.8	01	21	95.5%	< 0.0001*
Item 1.9	05	17	77.3%	< 0.0001*
Item 1.10	03	19	86.4%	0.0014*
Item 1.11	01	21	95.5%	< 0.0001*
Item 1.12	04	18	81.8%	0.0056*
Item 1.13	01	21	95.5%	< 0.0001*
Item 1.14	02	20	90.9%	0.0003*
Item 1.15	00	22	100.0%	< 0.0001*
Item 1.16	01	21	95.5%	< 0.0001*

EG = HTLV Experts Group.

*Significant result for the Chi-square Test of adherence.

The analysis using the Bland-Altman plot (Fig 1) showed agreement of the items in the content domain since the average of the differences observed in the CVI between the PG and EG failed to significantly differ from zero (p>0.05). The limits of agreement were also narrow (approximately 27%). Groups only disagreed in Item 1.9 as it showed values above the confidence interval (95%). The statement to be evaluated was ‘the messages are presented clearly and objectively’.

Although we found a sufficient index to deem the technology validated, we modified the booklet considering judges’ observations and suggestions, which we deemed relevant.

## Discussion

The validation process is relevant so that educational materials have no wrong or incomplete information that could mislead the target population or hinder their understanding of the theme [25]. HET must be written in a simple language with clear and precise information and attractive illustrations. The construction of the material should consider its content, language, organization, layout, illustrations and ways of learning [26–28]. The material should, rather than extensive, be objective, attractive, easy to understand, and meet the needs of the involved population. The use of illustrations is important both for a more attractive material and better understanding [24].

During the validation stage by judges, the CVI of the study technology ranged from 93.4 to 94.3%. This finding corroborates other methodological studies carried out in Brazil that obtained similar results, such as the validation of content with guidance on exercises for children with acute lymphocytic leukemia—with a CVI of 100% [29]—, the validation of a booklet to prevent metabolic syndrome in adolescents—with CVI of 100% in most analyzed items [30], the validation of an exercise program for women undergoing breast cancer surgery—with a CVI of 75% [31], the validation of an educational booklet on pelvic exercises for women with urinary incontinence—with CVI from 71 to 100% [32]—, and the validation of an educational booklet with physical exercises to prevent falls in older adults—with a CVI of 89% [24].

The groups of judges in this research consisted of multidisciplinary professionals, which, according to Alves [33] and Lima [24], is an advantage since practice faces great difficulty maintaining a single language regarding interdisciplinary guidance in health. The multidisciplinary approach is recommended to expand the content of educational material on the knowledge and experience of the different professionals involved in the process [34, 35].

Regarding gender, we obtained similar results to several others that involved validation steps [24, 33, 36], i.e., most evaluators were women, 60.9%. Regarding the higher education of evaluators, we observed that clinical practice increasingly requires stricto sensu courses, as they help professionals in decision-making, making them more capable of incorporating technologies that can assist in these situations [37].

Studies have used the home approach toward physical exercise due to its feasibility, long-term maintenance [28–31], and more accessible approach to exercise plans that can be performed at home and without special equipment. Home exercise allows removing the accessibility of training facilities [38], is economical [39–41] and reduces barriers to expenditure of locomotion time [39]. In addition, standardized home exercise protocols guided by socio-educational materials such as booklets have been identified as effective in the treatment of chronic degenerative diseases and in stimulating the individual’s autonomy to deal with their condition [42–48].

In a study carried out in the Brazilian state of Bahia [17], which evaluated the impact of a home exercise program with and without supervision on the functional mobility and pain of individuals with MAH/TSP, it was observed that, although there were several socioeconomic barriers experienced According to the population investigated, both modalities proved to be satisfactory in improving posture and functional mobility. Thus, the results found suggested that the use of an exercise booklet at home, with or without supervision, can alleviate several social and personal limitations encountered by people with HAM/TSP. Therefore, in situations of lack of vacancies in public physiotherapy services, the existence of obstacles regarding locomotion/transport or the lack of a companion when traveling to the rehabilitation service, the use of it autonomously can be applied.

Although it is impossible to standardize exercises and health professionals generally adopt verbal guidance, the use of written and audiovisual materials is important to reinforce verbal information and directly impact the effectiveness of teaching, facilitate the teaching-learning process and adherence to treatment, and serve as a guide and an aid to decision-making [33–35], it is necessary to create guidelines and encourage work on this research theme [32].

Considering the pedagogical concept of including subjects, the use of booklets as a health education technology highlights the humanist attitude of respect for differences, the acceptance of singularities, and equity in the opportunity of access to care for all. It is a palpable, timeless, easily accessible, and widely applicable resource to the target audience as it aims the home environment. Moreover, written communication can decode and assimilate information using clear language, using examples with illustrations and audiovisual resources, raising readers/patients’ critical awareness, guiding them toward a healthier life with greater autonomy, and thus leading to self-care [49].

Although a sufficient rate was achieved to consider the technology validated, modifications were made to the booklet, considering observations and suggestions from the judges, which were considered relevant. After adjustments, the booklet was sent back to the judges.

The limitations of this study include the convenience selection of judges and the technology assessment instrument not being validated, however, the restrictions are justified by the limited research on the development and validation of health education technologies aimed at coping with associated clinical manifestations to HTLV-1 that focus on the home approach, creating challenges in the development of exercises, and that consider all the criteria necessary for their good execution.

## Conclusions

The technology demonstrated is valid for use with the target audience, thus providing support in the prevention of functional losses in people living with HTLV-1, standardization of guidelines for physiotherapy professionals who accompany these patients and in the validation of exercise programs households with other comorbidities. It is recommended that this study continue with its application to this population, seeking to contribute to a safe, evidence-based home clinical practice and point out new paths in the production of health education technologies sensitive to the reality of this population.

## Supporting information

S1 File Final version of the technology. Final version of the technology called “Home Exercise Booklet for People Living with HTLV-1”.
(PDF)

S1 Data (XLSX)
