# Supplementary material for: Effectiveness assessment of a home-based exercise intervention in mitigating HTLV-1 associated disabilities: A validation study
Source: PLoS One. 2024 May 14;19(5):e0302542. doi: 10.1371/journal.pone.0302542 (PMC11093371; doi:10.1371/journal.pone.0302542)
Supplement: S1 File — Final version of the technology called “Home Exercise Booklet for People Living with HTLV-1”. (PDF) [file pone.0302542.s001.pdf]

CARTILHA DE EXERCÍCIOS DOMICILIARES PARA PESSOAS VIVENDO COM HTLV-1

Guia prático para realização de exercícios fisioterapêuticos em ambiente domiciliar direcionado a pessoas independentes vivendo com HTLV-1

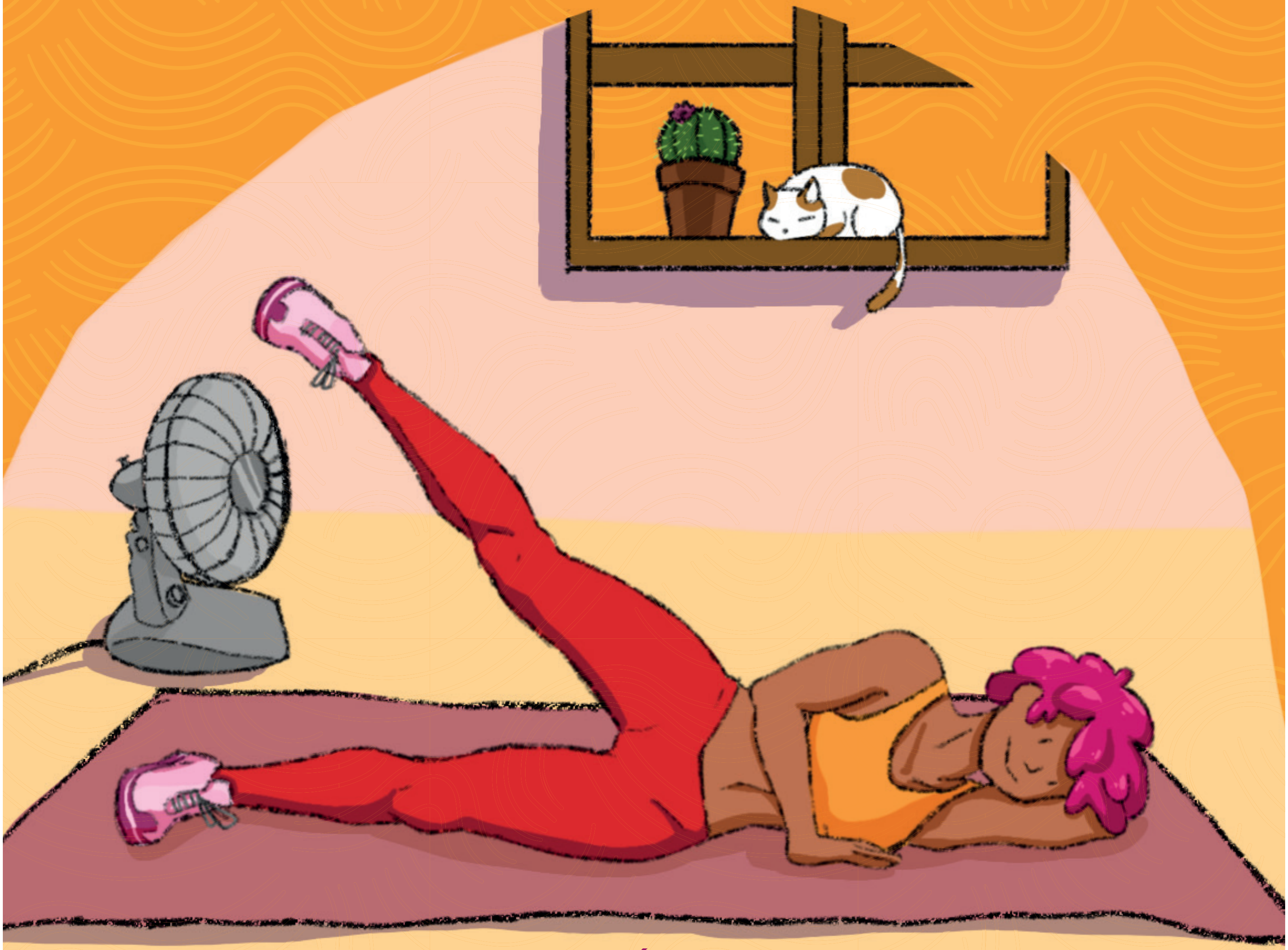

BELÉM-PA
2022

Esta Cartilha é parte integrante da tese de doutorado do Programa de Pós-Graduação em Doenças Tropicais (PPGDT), do Núcleo de Medicina Tropical – Universidade Federal do Pará (NMT/UFPA), intitulada "Desenvolvimento e Validação de uma Cartilha de Exercícios Domiciliares para contribuir na prevenção de perdas funcionais em pessoas vivendo com HTLV-1"
Obra Registrada na Fundação Biblioteca Nacional - Registro nº 872.080 em 31/03/2023.

Dados Internacionais de Catalogação na Publicação (CIP)
(Câmara Brasileira do Livro, SP, Brasil)

Assis, Izabela Mendonça de
Cartilha de exercícios domiciliares para pessoas vivendo com HTLV-1 [livro eletrônico] / Izabela Mendonça de Assis, Maísa Silva de Sousa, Bianca Callegari. -- Santarém, PA : Ed. das Autoras, 2023.
PDF

Vários colaboradores.
Bibliografia.
ISBN 978-65-00-68860-3

1. Doenças - Tratamento 2. Exercícios físicos - Métodos 3. Fisioterapia - Métodos 4. Saúde I. Sousa, Maísa Silva de. II. Callegari, Bianca. III. Título.

CDD-615.82
23-154630 NLM-WB-460

Índices para catálogo sistemático:

1. Fisioterapia : Reabilitação médica : Ciências médicas 615.82

Eliane de Freitas Leite - Bibliotecária - CRB 8/8415

FICHA TÉCNICA

Créditos

Universidade Federal do Pará - UFPA
Núcleo de Medicina Tropical
Programa de Pós Graduação em Doenças Tropicais

Autoras

Me. Izabela Mendonça de Assis - UFPA
Dra. Maísa Silva de Sousa - UFPA
Dra. Bianca Callegari - UFPA

Parceiros

Escola Bahiana de Medicina e Saúde Pública- EBMSP
Pesquisadoras do Grupo de Pesquisa Dinâmica do Sistema Neuromusculoesquelético da Escola Bahiana, Linha de Pesquisa HTLV: Katia Nunes Sá, Selena Dubois e Maíra Carvalho Macêdo

Colaboradores

Ilustrações: Brunna Sanae Shiya Silva e Débora Marcião Santos - Estúdio Taberna e Vanessa Karoline Campos Rêgo
Filmagem: Carlos de Matos Bandeira Junior - Bandeira Filmes
Edição de vídeos: Ilgner Juan dos Santos Lima
Tradução em Libras: Kellen Maria Garcia de Sousa
Diagramação: Andressa Fernanda Pedroso Azevedo

APRESENTAÇÃO

Prezado(a) Leitor(a),

Esta cartilha descreve um programa de exercícios de Fisioterapia adaptado para o ambiente domiciliar e tem como finalidade oportunizar a prática regular de exercícios em casa e contribuir na prevenção de declínios funcionais.

O objetivo deste programa é permitir que você desempenhe cada vez mais e melhor as suas atividades da vida diária, como caminhar, vestir-se, tomar banho, subir e descer escadas e controlar a urina. Também busca contribuir em seu equilíbrio e na sua postura, assim como na forma como caminha.

Esperamos que este programa contribua para a melhoria de sua condição de saúde e proporcione uma maior motivação para manter suas atividades cotidianas, colaborando na sua qualidade de vida.

Você poderá ver e ouvir essa cartilha acessando pelo QR-CODE o canal no youtube.

Cartilha de Fisioterapia HTLV-1

SUMÁRIO

Começando nossa conversa! 05

Conheça mais sobre o HTLV 06

Prevenção e Tratamento 09

Afinal, por que praticar exercícios físicos? 10

Para quem a cartilha é direcionada? 12

Onde este PDE se encaixa no Tratamento Fisioterapêutico? 12

Conselhos, Orientações 13

Frequência e Prescrição dos Exercícios 14

Materiais necessários para evolução dos exercícios 16

Programa Domiciliar de Exercícios 17

Curiosidades 30

Agradecimentos 31

Referências 32

Diário de Exercícios 34

Seguindo estas orientações, você poderá ter uma qualidade de vida melhor, poderá prevenir incapacidades na realização de atividades da vida diária e complicações associadas ao HTLV-1, que podem e devem ser evitadas.

A vida é a soma de todas as suas escolhas.

Albert Camus

FISIOTERAPIA: estratégia e ação com movimento inteligente para potencializar a funcionalidade humana.

Luciana Bilitário

COMEÇANDO NOSSA CONVERSA!

O Vírus T-Linfotrópico Humano (HTLV) é um vírus que infecta determinadas células do sangue (linfócitos T). Pertence à família dos retrovírus e tem ampla distribuição mundial, com estimativa de aproximadamente 20 milhões de pessoas contaminadas.

Originalmente descoberto nos Estados Unidos em 1980, o HTLV tem sido encontrado nos dias de hoje no sudoeste do Japão, ilhas do Caribe, África Equatorial e a América do Sul, incluindo o Brasil.

Confira então quais são os tipos de HTLV, seus sintomas e se existem tratamentos para esta condição de saúde.

CONHEÇA MAIS SOBRE O HTLV

Os tipos virais mais difundidos em todo o mundo são o **HTLV-1** e o HTLV-2. Em ambos os tipos, as pessoas, em sua maioria, permanecem assintomáticas ao longo da vida.

O primeiro está relacionado a doenças neurais graves e degenerativas, como a Paraparesia Espástica Crônica conhecida por Paraparesia Espástica Tropical/ Mielopatia associada ao HTLV-1- HTLV-1 (PET/MAH) ou também chamada pelo termo em inglês de HTLV-1 *Associated Myelopathy/Tropical Spastic Paraparesis* (**HAM/TSP**). Este também apresenta doenças sanguíneas, como a Leucemia/ Linfoma de Células T do Adulto (**ATL**).

Inflamações progressivas dos músculos, das articulações, dos olhos e da pele são condições que também estão relacionados com esse primeiro tipo viral.

O HTLV-2 não tem suas consequências tão bem claras e definidas, afinal, ele quase nunca provoca qualquer dano ao organismo infectado, sendo pouco associado a doenças.

Além destes, outros dois tipos virais foram relatados na África (HTLV-3 e HTLV-4).

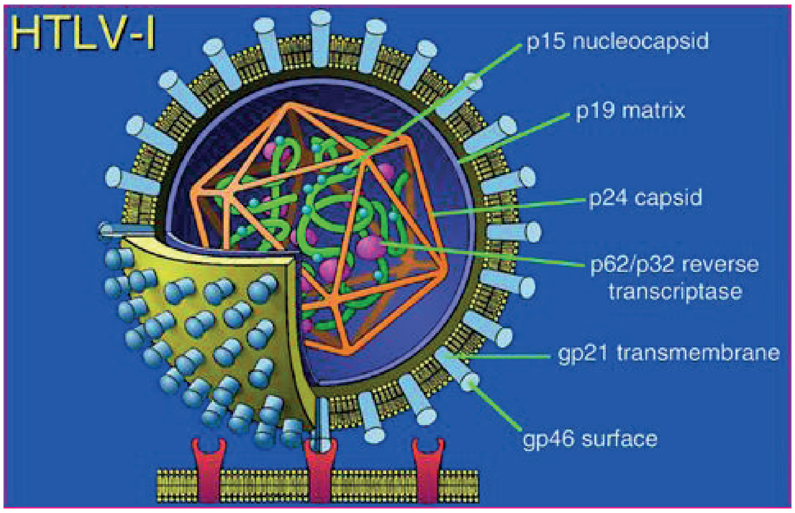

Vírus T-Linfotrópico Humano 1

Foto cortesia de AMH Nationwide no Flickr

Transmissão do vírus

As formas de transmissão do HTLV são:

- Relação sexual desprotegida com uma pessoa infectada pelo vírus;
- Transfusões de sangue e transplante de órgãos. Porém, a partir de 1993, o governo brasileiro tornou obrigatório o teste de triagem nos bancos de sangue para HTLV-1, o que gera mais segurança atualmente aos receptores após essa data;
- Compartilhamento de seringas, agulhas e/ou outros materiais que cortem ou perfurem infectados;
- Da mãe infectada para a o recém-nascido (principalmente através da amamentação. As evidências são limitadas na transmissão intrauterina ou durante o parto).

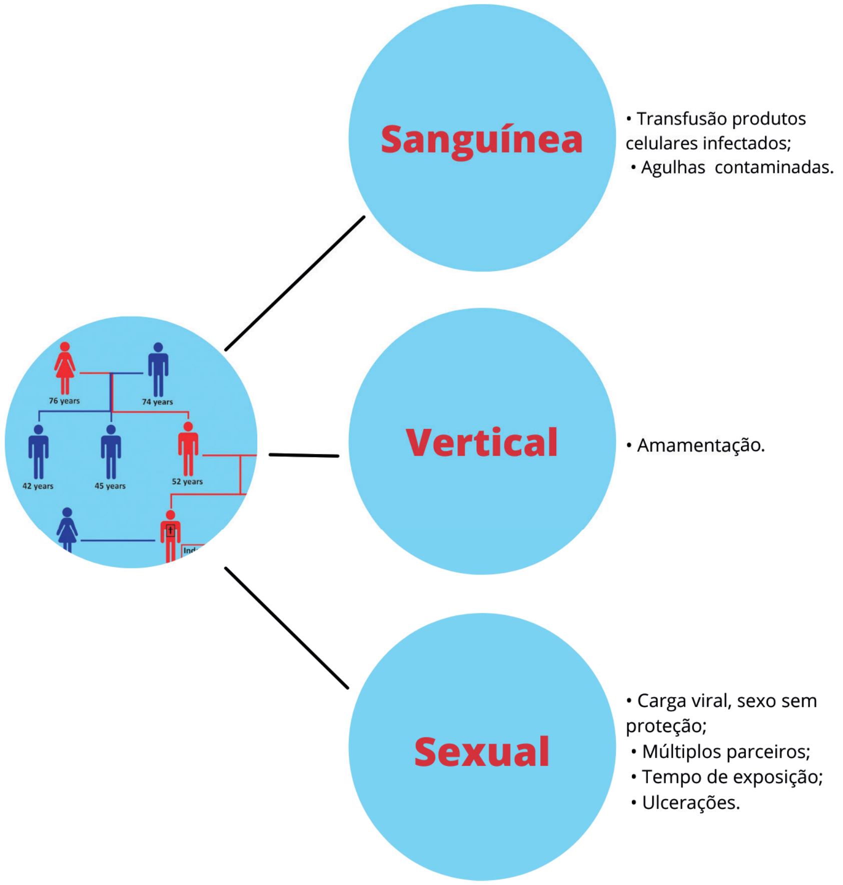

Fonte: Manejo Multidisciplinar de pessoas vivendo com HTLV. Casseb (2020) com adaptações da autora.

Quais são os sintomas?

Embora o HTLV-1 apresente uma baixa taxa de pessoas que apresentem doenças associadas, estudos científicos atuais têm observado bastantes sintomas neurológicos, além das doenças clássicas associadas ao vírus, como: sensação de dormência nas mãos e nos pés, incontinência e retenção urinárias e dificuldades para andar ou realizar atividades do dia a dia. Isso ocorre porque por muitos anos a doença é silenciosa, sem manifestar sintomas, ou estes são leves, podendo passar despercebidos. No entanto, os sintomas são progressivos, podendo levar a pessoa a necessitar do uso de meios auxiliares de locomoção, como muletas, andadores ou cadeira de rodas.

Outras síndromes neurológicas podem estar associadas à paraparesia espástica tropical ou ocorrer isoladamente no indivíduo infectado pelo HTLV-1. Perda de memória, problemas cerebrais, falta do controle da bexiga urinária, doenças neurológicas também podem ocorrer no paciente infectado pelo HTLV-1 e podem passar despercebidas ao profissional de saúde pouco familiarizado ao tema. Por isso, reforça-se a importância de um acompanhamento clínico, neurológico e funcional, com intervenção imediata, sendo uma delas a realização de exercícios.

A infecção pelo HTLV-1 é uma doença neurológica crônica, progressiva, insidiosa, tem maior incidência em mulheres e o surgimento dos sintomas tende a aparecer entre a quarta e quinta década de vida. Quando estes sintomas surgem, são geralmente sinais de alerta para procura de uma avaliação neurológica:

- Dores na panturrilha e nos pés;
- Dores na coluna lombar;
- Fraqueza muscular;
- Dormência e formigamentos nas pernas;
- Diminuição da sensibilidade nas pernas;
- Fadiga

- Dor neuropática (tipo de dor crônica que ocorre quando os nervos sensitivos do Sistema Nervoso Central e/ou Periférico são feridos ou danificados)
- Problemas urinários (como não segurar a urina, conhecida por incontinência)

Podem ocorrer manifestações graves da infecção, como alguns tipos de câncer, leucemias e linfomas, além de problemas musculares, nas articulações (artropatias), nos pulmões, na pele (dermatites), oftalmológicos e urológicos.

Nos quadros em que ocorrem leucemia e/ou cânceres do sistema de combate a infecções, conhecido em nosso corpo como sistema linfático. Os sintomas mais comuns são:

- Descamações e lesões graves na pele;
- Gânglios infartados, como exemplo as ínguas, que surgem quando nosso organismo está combatendo alguma infeção;
- Problemas visuais, como inflamações de partes que compõem nossos olhos.

PREVENÇÃO E TRATAMENTO

Assim como as diversas outras infecções sexualmente transmissíveis (**IST's**), o uso de preservativos é fundamental em todas as relações sexuais, além do não compartilhamento de seringas/agulhas ou qualquer outro objeto cortante.

Para as gestantes, recomenda-se a realização do rastreio da infecção no pré-natal para uma gravidez saudável e sem riscos e com um parto muito bem planejado. Para lactantes que vivem com o HTLV-1, recomenda-se a não amamentação.

No que diz respeito ao tratamento sintomático da fraqueza muscular, tecnicamente conhecida como paresia, é indicada a realização de Fisioterapia para o fortalecimento muscular dos braços, pernas e do tronco, melhora do equilíbrio e da amplitude das articulações, desenvolvimento da qualidade do andar e, em pacientes com mobilidade reduzida, é indicada a terapia ocupacional,

área responsável por promover a saúde e bem-estar das pessoas com problemas físicos, sensoriais, sociais e motores.

Quanto ao tratamento sintomático da espasticidade, sintoma que pode produzir dificuldades funcionais, deformidades musculares e dores, é indicado o uso de relaxantes musculares ou toxina botulínica, conhecida popularmente como **Botox**.

As manifestações neurológicas do HTLV-1 ainda não apresentam consenso na literatura acerca da existência de um tratamento específico comprovadamente eficaz. Porém, nestas circunstâncias, indica-se o encaminhamento para centros especializados de assistência à pessoa com HTLV-1 com equipe multiprofissional, como:

- **Núcleo de Medicina Tropical vinculado à Universidade Federal do Pará (NMT/UFPA) localizado em Belém-PA;**
- Instituto de Infectologia Emílio Ribas, com sede em São Paulo-SP;
- Ambulatório Especializado para Tratamento do HTLV, em Salvador-BA.

Uma pessoa que esteja com a infecção deverá procurar equipe multiprofissional especializada para ser examinada o quanto antes. Isso possibilita a detecção precoce de qualquer sinal de doença e iniciar os tratamentos necessários.

Quanto mais cedo forem tratadas as complicações causadas pelo HTLV-1, maiores são as chances de a pessoa ter qualidade de vida.

AFINAL, POR QUE PRATICAR EXERCÍCIOS FÍSICOS?

Sabe-se que alterações do movimento causadas por doenças que limitam fazer atividades sociais, laborais e/ou de vida diária são consideradas de risco à saúde.

O Fisioterapeuta desempenha um papel fundamental nessas situações,

estabelecendo o diagnóstico de acordo com as limitações individuais e o planejamento de um programa de reabilitação. Os programas de exercícios frequentemente são prescritos para amenizar ou reverter alterações motoras e neurológicas.

Pesquisas científicas têm demostrado que a aplicação de programas de fortalecimento muscular voltados a atividades funcionais revelaram melhora nas medidas de performance funcional de populações acometidas por agravos neurológicos. Com base nesses achados, sugere-se que os exercícios físicos constituem uma modalidade para melhora da funcionalidade de pessoas independentes vivendo com HTLV-1.

Para a Organização Mundial de Saúde - OMS (2011), a funcionalidade e a incapacidade de uma pessoa são entendidas como uma interação dinâmica entre as condições de saúde (doenças, lesões e deficiência) e os fatores contextuais (fatores ambientais e pessoais). Portanto, o processo de reabilitação exige estratégias de prevenção e de apoio, a fim de permitir que estas pessoas possam atingir e manter um nível ótimo de funcionalidade enquanto interagem com o seu ambiente.

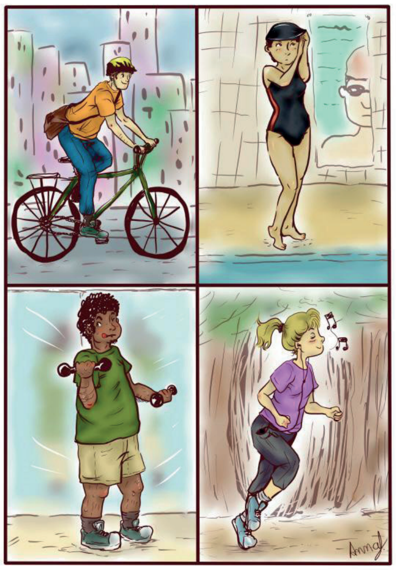

Atividade Física

Foto cortesia de Anna Jonko no Flickr.

PARA QUEM A CARTILHA É DIRECIONADA?

Este programa de exercícios é direcionado àquelas pessoas que recentemente foram comunicadas que vivem com HTLV-1 ou que já sabem, porém, apresentam sintomas motores leves associados à infecção.

Ressalta-se que a Fisioterapia é um dos recursos indicados no tratamento de pessoas vivendo com HTLV-1 e somente pode ser aplicado por um profissional habilitado, com conhecimentos sobre a fisiopatologia da doença (os mecanismos que explicam seus sintomas), sobre controle motor (como o sistema nervoso organiza seus movimentos) e sobre cinesiologia (ciência que estuda os movimentos). Com o domínio de todas essas informações, o fisioterapeuta tem condições de avaliar o caso de cada paciente e elaborar um programa de tratamento específico.

ONDE ESTE PROGRAMA DOMICILIAR DE EXERCÍCIOS SE ENCAIXA NO TRATAMENTO FISIOTERAPÊUTICO?

Este programa tem como finalidade auxiliar pessoas, familiares e cuidadores na luta contra os sintomas motores da infecção pelo HTLV-1. Ele é resultado de um extenso levantamento de evidências científicas sobre programas domiciliares de exercícios e sintomas motores associados à infecção pelo HTLV-1 para o desenvolvimento e validação desta tecnologia de educação em saúde.

Vale ressaltar que este programa não tem a pretensão de substituir o trabalho do profissional fisioterapeuta, muito menos difundir entre as pessoas vivendo com o vírus que podem se tratar em casa por conta própria.

Assim, este programa é dedicado a um grupo de pessoas que podem ser

classificadas como independentes, ou seja, apresentam sintomas motores leves ou são assintomáticas, são capazes de realizar quase ou todas as atividades motoras da vida diária, podendo sentir apenas alguma diminuição da força muscular, principalmente nas pernas.

Diante desse quadro, a principal preocupação deste programa é a manutenção da força muscular para a realização de suas atividades cotidianas.

CONSELHOS GERAIS

- Realize os exercícios com roupa confortável e em ambiente fresco e arejado de sua casa, se possível na frente de um espelho, como na sala, na área externa etc;
- Durante o treinamento, a carga dos exercícios NÃO deve ser percebida como MUITO DIFÍCIL, exemplo quando não conseguir executar o exercício por motivo de força muscular, dor ou condicionamento físico;
- As repetições dos movimentos são importantes da mesma maneira que é importante o descanso;
- Os exercícios não devem causar dor, cãibras ou cansaço excessivo. Ao sentir qualquer um desses sintomas, faça uma pausa. Se tais sintomas forem frequentes, informe à equipe multiprofissional que realiza seu acompanhamento.

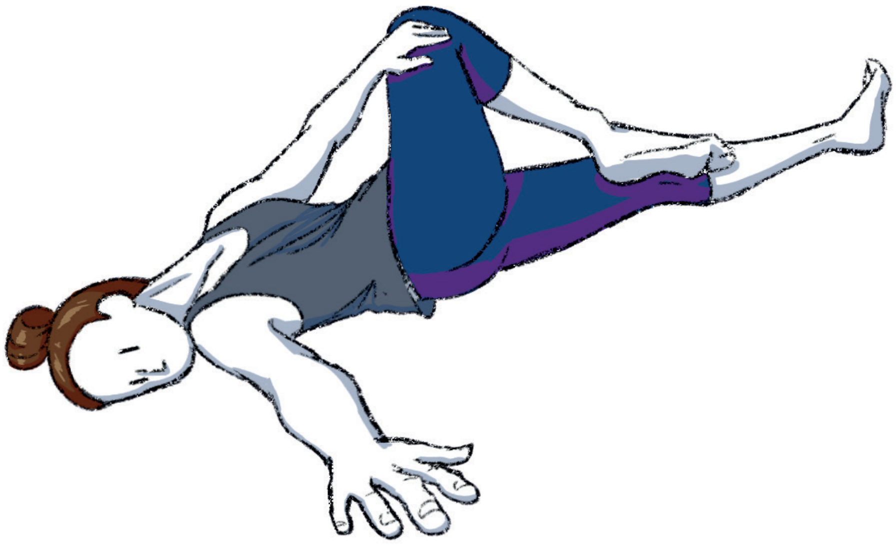

ORIENTAÇÕES PARA OS EXERCÍCIOS

- Realize os exercícios de forma lenta e controlada, mantendo sempre a postura correta;
- Durante a realização dos exercícios, evite prender a respiração e, procure "soltar o ar" enquanto executa os movimentos;
- Treine sempre os lados direito e esquerdo do corpo, mesmo que a figura só mostre um lado. Somente alterne o lado após finalizar o número total das séries;

OBS: Lembre-se que **série** equivale a um bloco/conjunto de repetições de exercícios e **repetição** é o número de exercícios praticados dentro da série.

- Descanse pelo menos um minuto entre uma série e outra;
- Siga corretamente a ordem dos exercícios mostrada na cartilha;
- Após a finalização, marque os dias da semana que realizou os exercícios em um DIÁRIO DE EXERCÍCIOS e registre as modalidades de exercícios executados (alongamento e/ou fortalecimento), além de observações que ache pertinente.
- Comparecer a consultas de rotina e realizar acompanhamento se possível com equipe multidisciplinar.

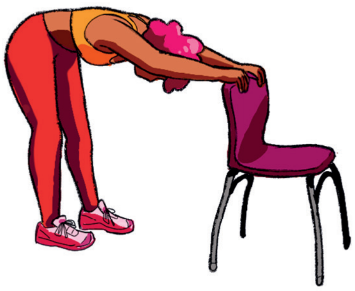

FREQUÊNCIA DOS EXERCÍCIOS

- Realize os exercícios 3 vezes por semana, com pelo menos intervalo de 1 dia entre eles;
- Leia as instruções, observe atentamente as figuras de cada exercício e acompanhe os vídeos de execução.

PROGRESSÃO DOS EXERCÍCIOS

O Programa Domiciliar de Exercícios - PDE é composto por seis exercícios de alongamento e dez exercícios de fortalecimento muscular, envolvendo os membros superiores, membros inferiores e a região do tronco. A prescrição de todos os parâmetros dos exercícios se manterá inalterada ao longo de todo o programa (quadro 1), exceto a intensidade, onde está previsto o aumento periódico da resistência através do aumento do número de repetições, de acordo com a disposição e desempenho de cada pessoa para cada grupamento muscular.

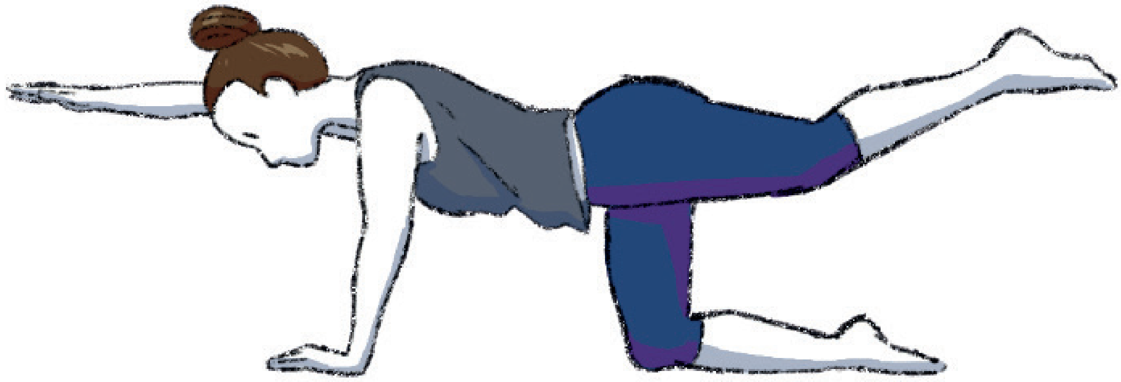

Parâmetros utilizados para a prescrição dos exercícios de alongamento e fortalecimento muscular

PARÂMETROS	ALONGAMENTO	RESISTIDOS
**Frequência**	3 vezes por semana	3 vezes por semana
**Duração**	20 a 30 segundos	Mínimo 5 repetições<br>Máximo 10 repetições
**Número de séries**	3	3
**Intensidade**	Sensação de leve desconforto	Mínimo 5 repetições<br>Máximo 10 repetições
**Intervalo**	1 minuto	1 minuto
**Progressão**	-	Aumento gradativo no número de repetições conforme a disposição.

MATERIAIS NECESSÁRIOS

Os materiais necessários para a realização dos exercícios em casa são:

- 1 toalha ou 1 lençol fino/pequeno;
- 1 cadeira;
- 1 bastão ou cabo de vassoura;
- 1 tapete antiderrapante ou módulos de EVA;

OBS: EVA é um material antiderrapante emborrachado e flexível indicado para diversas atividades físicas.

- 1 celular com acesso à internet para o acompanhamento dos vídeos explicativos;
- 1 cuidador/familiar para acompanhamento dos exercícios em execução para aqueles que principalmente apresentem mobilidade reduzida.

Qualquer dúvida, entre em contato com a Fisioterapeuta responsável pelo Programa Domiciliar de Exercícios, Izabela Mendonça: **(93) 98129-1212** ou com o Laboratório de Biologia Molecular e Celular/Núcleo de Medicina Tropical/UFPA **(91) 3201-0960**. Endereço eletrônico: izabela_stm@hotmail.com

CARTILHA DE EXERCÍCIOS DOMICILIARES PARA PESSOAS VIVENDO COM HTLV-1

A execução de todos os exercícios você pode acompanhar em nosso canal no Youtube:

Cartilha de Fisioterapia HTLV-1

Exercícios de Alongamento

Os alongamentos permitem que seu corpo fique mais preparado para aguentar as tensões que atividades físicas trazem, pois aumenta a amplitude de movimento dos músculos, evitando que a sobrecarga cause lesões e dores em outras partes do corpo. Leia com atenção as instruções!

1. ALONGAMENTO DA COLUNA LOMBAR

Este exercício tem como objetivo aliviar a tensão muscular e, por consequência, a dor lombar, além de preparar o corpo para os exercícios seguintes.

Deitado, abrace os joelhos por cima da perna e puxe-os lentamente em direção ao tórax. Tente manter o alongamento localizado na coluna lombar e evite distender o pescoço ou os ombros. Sustente essa posição durante 30 segundos e repita pelo menos 3 vezes.

Exercício 1

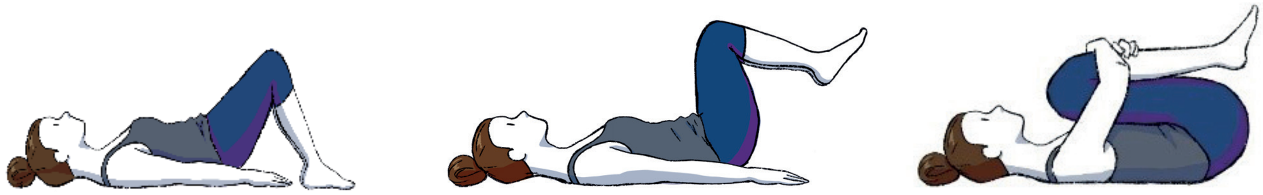

2. ALONGAMENTO DOS MÚSCULOS POSTERIORES DA COXA

Este exercício é importante para evitar uma possível tensão na região, que pode se estender para outras partes do corpo e provocar dores nas costas.

Deitado, eleve uma das pernas, mantendo o joelho esticado e sustente com uma toalha ou lençol até sentir esticar o músculo. Repita o mesmo procedimento com a outra perna 3 vezes, sustentando 30 segundos cada vez.

Exercício 2

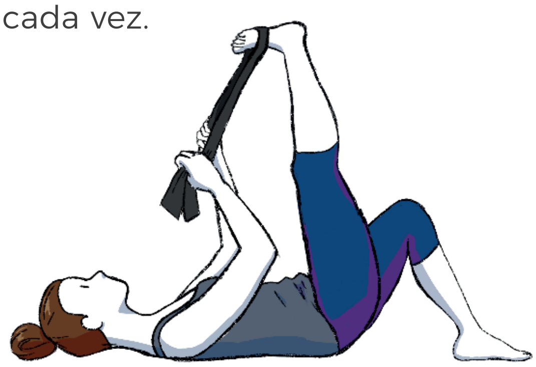

3. BORBOLETA – ALONGAMENTO DOS MÚSCULOS INTERNOS DA COXA

Este exercício é eficaz para aliviar a tensão nos quadris e aumentar a flexibilidade, especialmente após movimentos repetitivos ou ficar sentado por muito tempo.

Sentado, dobre os joelhos até que as solas de ambos os pés se encontrem. Incline o tronco para frente, na direção dos pés. Sustente essa posição durante 30 segundos e repita 3 vezes.

Exercício 3

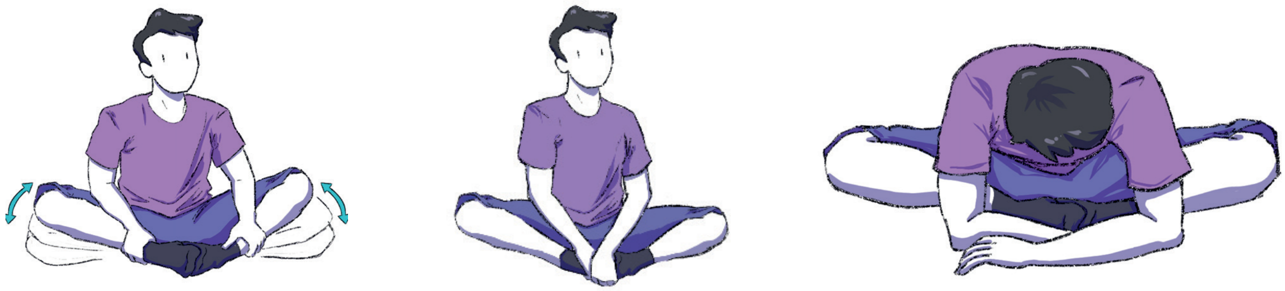

4. AMARRA CADARÇO – ALONGAMENTO DOS MÚSCULOS ROTADORES EXTERNOS DO QUADRIL E POSTERIORES DO TRONCO

Este exercício contribui na melhora da flexibilidade do quadril para realização de atividades como amarrar cadarço sem dificuldade.

Sentado, dobre uma das pernas sobre a outra e tente alcançar os seus pés. Durante 3 vezes, sustente essa posição por 30 segundos. Repita a postura com a outra perna.

Exercício 4

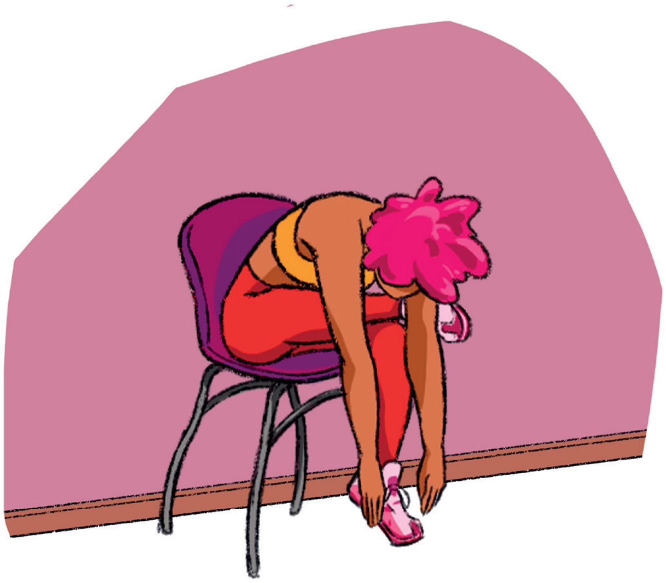

5. XÔ PREGUIÇA - ALONGAMENTO DOS MÚSCULOS LATERAIS DO TRONCO

Este exercício ajuda a corrigir a postura e realinhar a musculatura e se trata de um exercício importantíssimo, principalmente, se você passa muito tempo em frente ao computador ou trabalha sentado(a).

Sentado, com as pernas afastadas e os pés firmes no chão, estique os braços para cima e cruze os dedos das duas mãos entre si. Incline lateralmente o tronco de um lado e permaneça nessa posição por 30 segundos. Repita a posição para o lado contrário.

Exercício 5

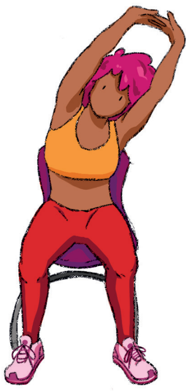

6. SACI-PERERÊ - ALONGAMENTO DOS MÚSCULOS ANTERIORES DA COXA

Este exercício ajuda a prevenir lesões que podem ocorrer no joelho.

Em pé, apoiada(o) em uma cadeira ou parede, puxe o peito do pé em direção às nádegas/bumbum. Procure manter a coxa alinhada com o tronco, evitando inclinação para trás ou para os lados. Durante 3 vezes, sustente essa posição por 30 segundos e repita o mesmo procedimento com a outra perna.

Exercício 6

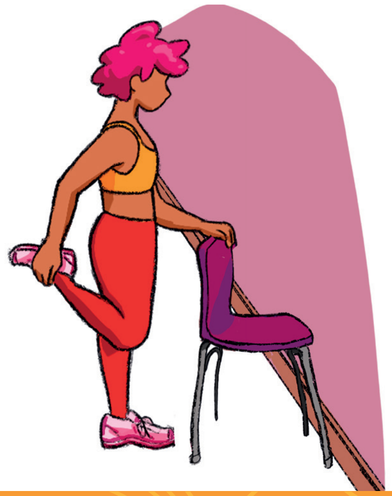

Exercícios de Mobilidade e Fortalecimento

Os exercícios descritos a seguir tem como finalidade prevenir a fraqueza muscular ao caminhar, correr, carregar e alcançar objetos, tomar banho, vestir-se e controlar a urina. Fique atenta(o) às instruções!

7. NA PONTA DOS PÉS – EXERCÍCIO DE AQUECIMENTO

Este exercício ajuda a manter o equilíbrio das pernas. As "batatas" estão entre os locais que mais recebem sangue no corpo, tendo papel fundamental para a circulação em nosso organismo. Assim, é necessária atenção total à musculatura, mantendo sempre forte e tonificada.

Em pé, fique na ponta dos pés, apoiando seus braços em uma superfície. Repita por 10 vezes durante 3 séries de 10.

Exercício 7

Séries: 3 **Repetições:** 10 **Intervalo de descanso:** 1 min

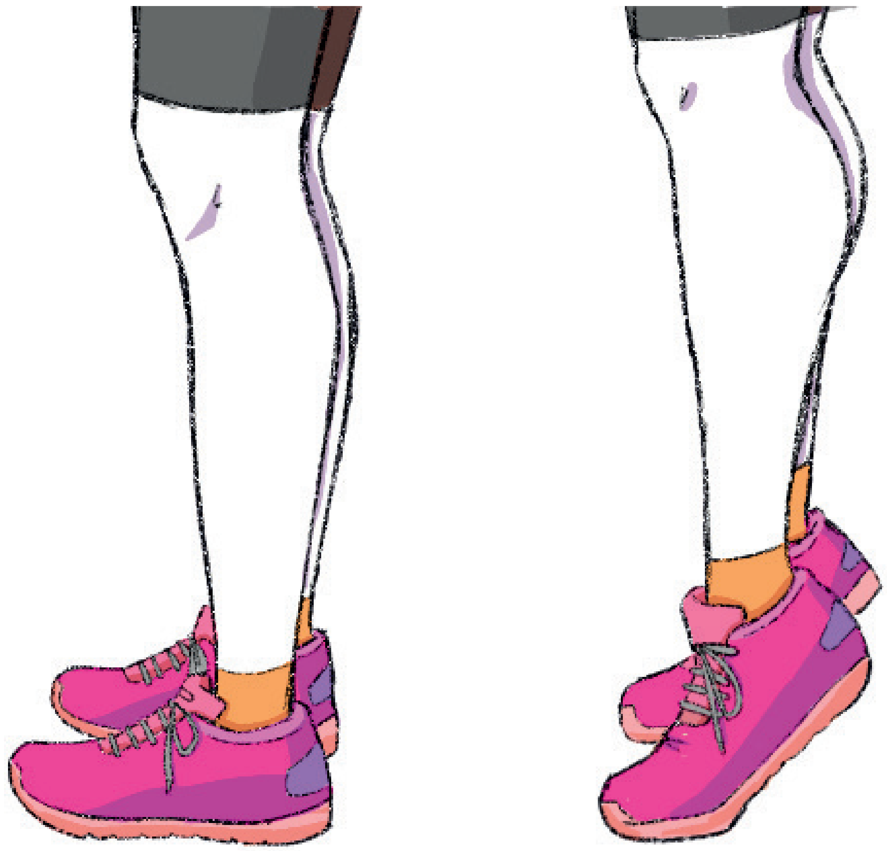

8. AGACHAMENTO - EXERCÍCIO DE AQUECIMENTO

Este exercício é eficaz para fortalecer e desenvolver os músculos das coxas, pernas e bumbum. Auxilia na melhora na mobilidade do quadril, das pernas, do equilíbrio e do desenvolvimento da qualidade de seu andar.

Em pé, afaste os pés na direção dos seus ombros mantendo os joelhos apontados para fora. A seguir, simule "sentar" em um banco direcionando o seu quadril para trás, mantendo os braços esticados para frente na altura dos seus ombros. Faça 10 repetições em 3 séries.

Séries: 3 **Repetições:** 10 **Intervalo de descanso:** 1 min

Exercício 8

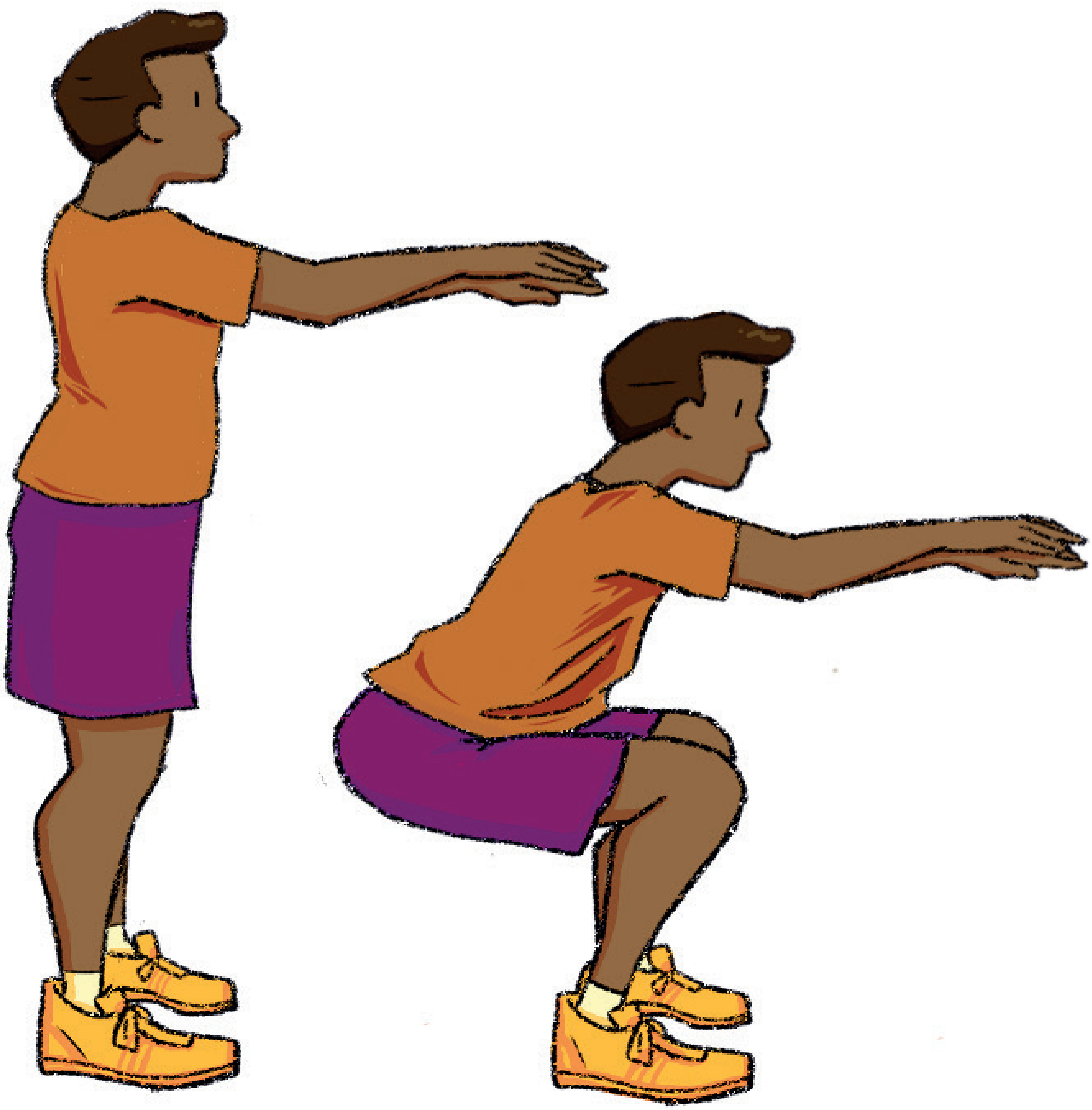

9. REMADA CONJUGADA – FORTALECIMENTO DOS MÚSCULOS DO TRONCO E OMBROS

Este exercício atua principalmente na musculatura das costas e dos ombros, além de também atuar como um ótimo exercício de condicionamento físico, já que o corpo se cansa depois de algumas repetições.

Neste exercício, você vai precisar utilizar um bastão. Sentado, com a coluna bem apoiada e as mãos voltadas para frente, mova o bastão para cima e retorne à posição na altura dos ombros. Em seguida, mova o bastão para frente, estendendo os cotovelos, simulando uma remada e retorne à posição na altura dos ombros. Repita por 10 vezes em 3 séries.

Exercício 9

Séries: 3 **Repetições:** 10 **Intervalo de descanso:** 1 min

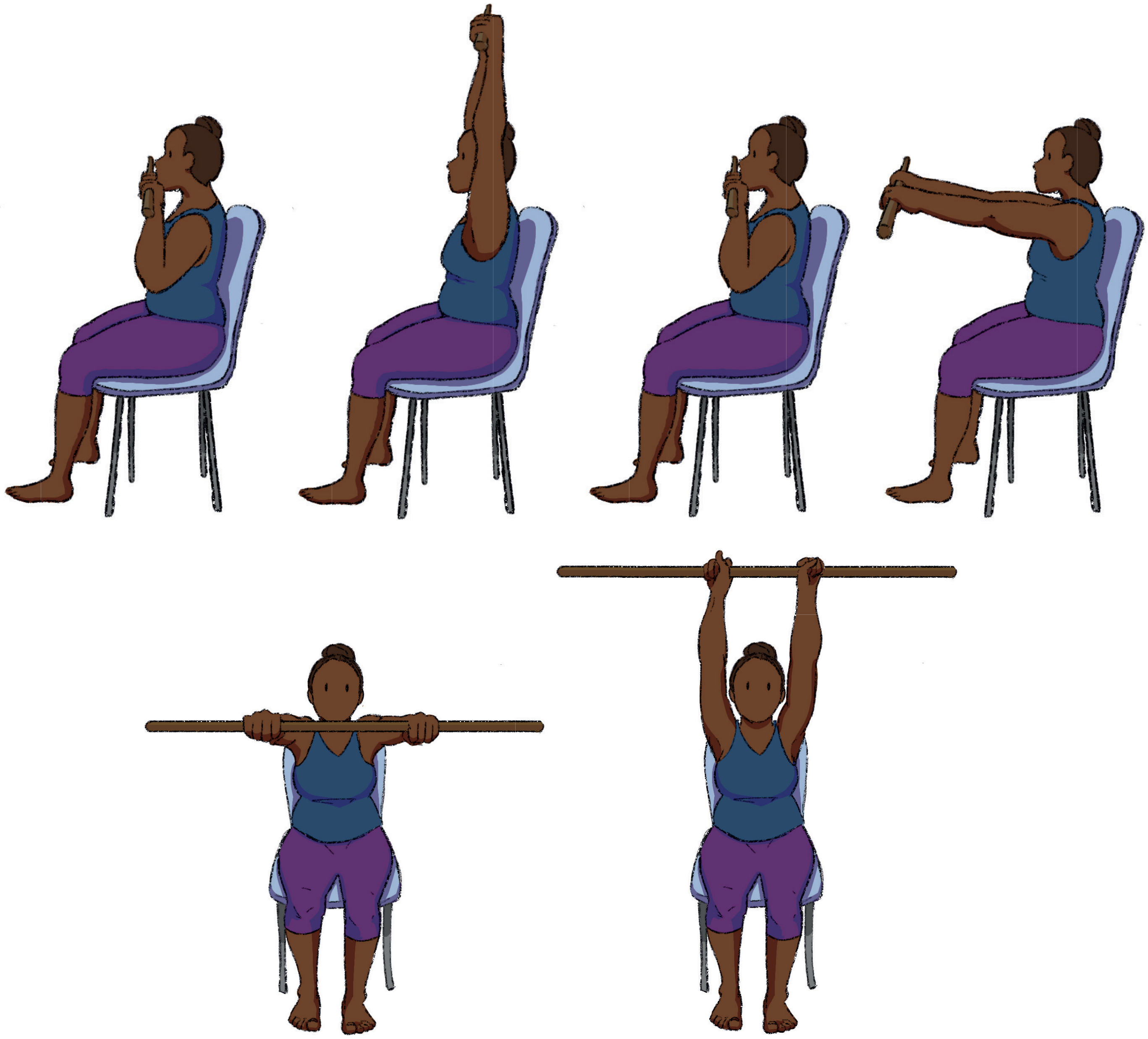

10. AGACHAMENTO – EXERCÍCIO DE AQUECIMENTO

Este exercício é indicado para fortalecer a coluna, os músculos das pernas, o bumbum e controlar a urina.

Deite-se em um colchonete ou tapete, com os braços esticados ao longo do corpo e joelhos dobrados com os pés no chão. Com o abdômen e o bumbum (glúteo) contraídos, levante o quadril o máximo que puder, contraindo os músculos que seguram a urina (assoalho pélvico). Desça devagar até tocar o quadril no chão e relaxe a musculatura do assoalho pélvico. Faça entre 10 e 15 repetições em 3 séries. Descanse 1 minuto.

Exercício 10

Séries: 3 **Repetições:** 10 a 15 **Intervalo de descanso:** 1 min

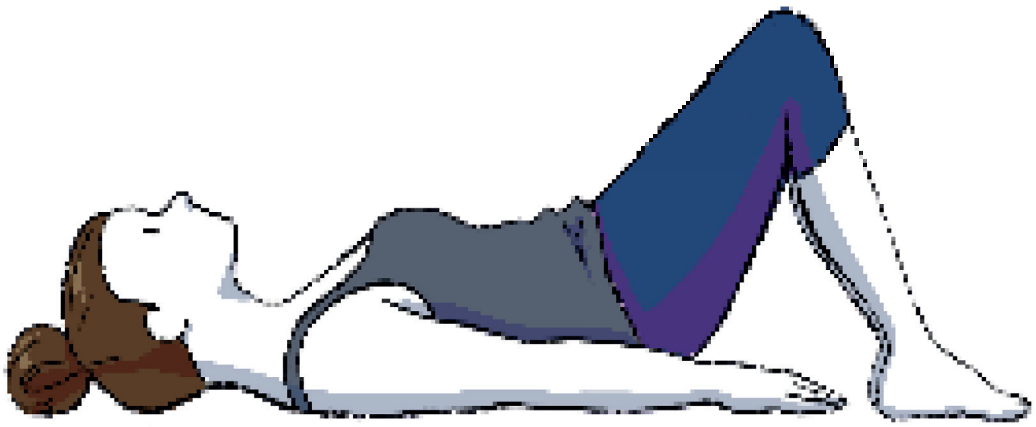

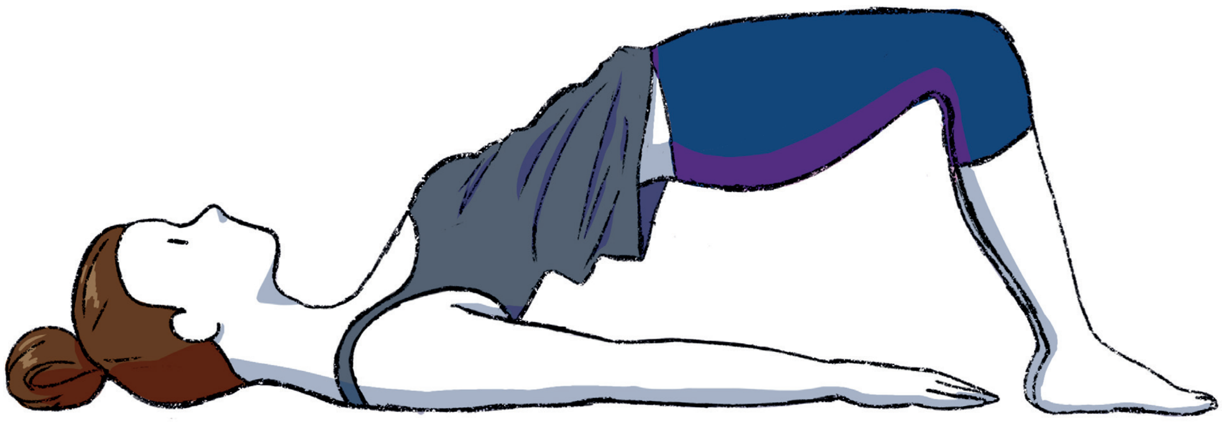

11. BICICLETA NO AR – FORTALECIMENTO DOS MÚSCULOS ABDOMINAIS

Este exercício é uma variação do exercício abdominal e ótima opção para queimar calorias e fortalecer os músculos, tanto do abdômen quanto das pernas.

Deitado de barriga para cima, coloque as mãos ao lado do corpo, eleve as pernas e faça o movimento de bicicleta, como se estivesse pedalando no ar. Para os iniciantes, fique até 20 segundos nessa posição. Para quem tem condicionamento físico, fique até 1 minuto.

Séries: 3 **Repetições:** 20 a 60 seg **Intervalo de descanso:** 1 min

Exercício 11

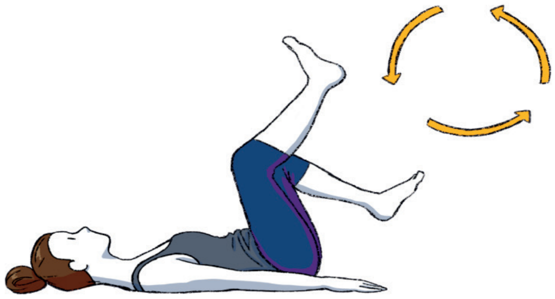

12. LIMPADOR DE PARA-BRISA – FORTALECIMENTO DOS MÚSCULOS INTERNOS DA COXA

Este exercício ajuda na mobilidade do quadril, aumenta a eficiência em corridas ou caminhadas em maior velocidade e melhora a capacidade física em atividades cotidianas.

Deitado, com joelhos esticados e pernas apoiadas na parede, abra-as em 90 graus e separe-as lateralmente o máximo que puder. Em seguida, volte a juntá-las na posição de partida. Faça isso 10 vezes em 3 séries, com descanso de um minuto.

Séries: 3 **Repetições:** 10 a 15 **Intervalo de descanso:** 1 min

Exercício 12

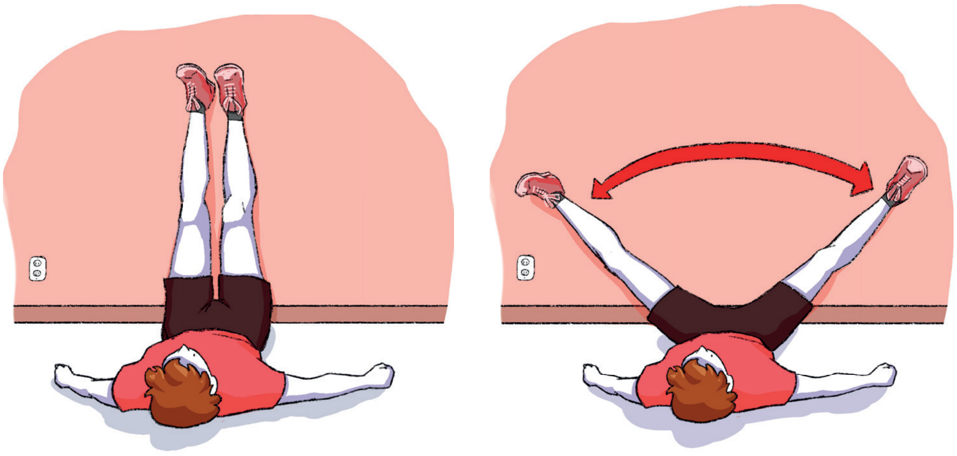

13. ELEVAÇÃO LATERAL DA PERNA – FORTALECIMENTO DA ARTICULAÇÃO DO QUADRIL

Neste exercício é possível trabalhar um pouco do abdominal lateral, do bumbum e, principalmente, a região da coxa, sendo uma excelente opção para perda calórica.

Deite-se de lado, encostado em uma parede. A perna mais próxima ao chão deve estar levemente dobrada e a perna superior deve estar reta. Levante lentamente a perna superior, mantendo o joelho reto e os dedos dos pés apontados para a frente. Repita o mesmo procedimento com a outra perna. Faça isso 10 vezes em 3 séries, com descanso de um minuto em cada perna.

Séries: 3 **Repetições:** 10 **Intervalo de descanso:** 1 min

Exercício 13

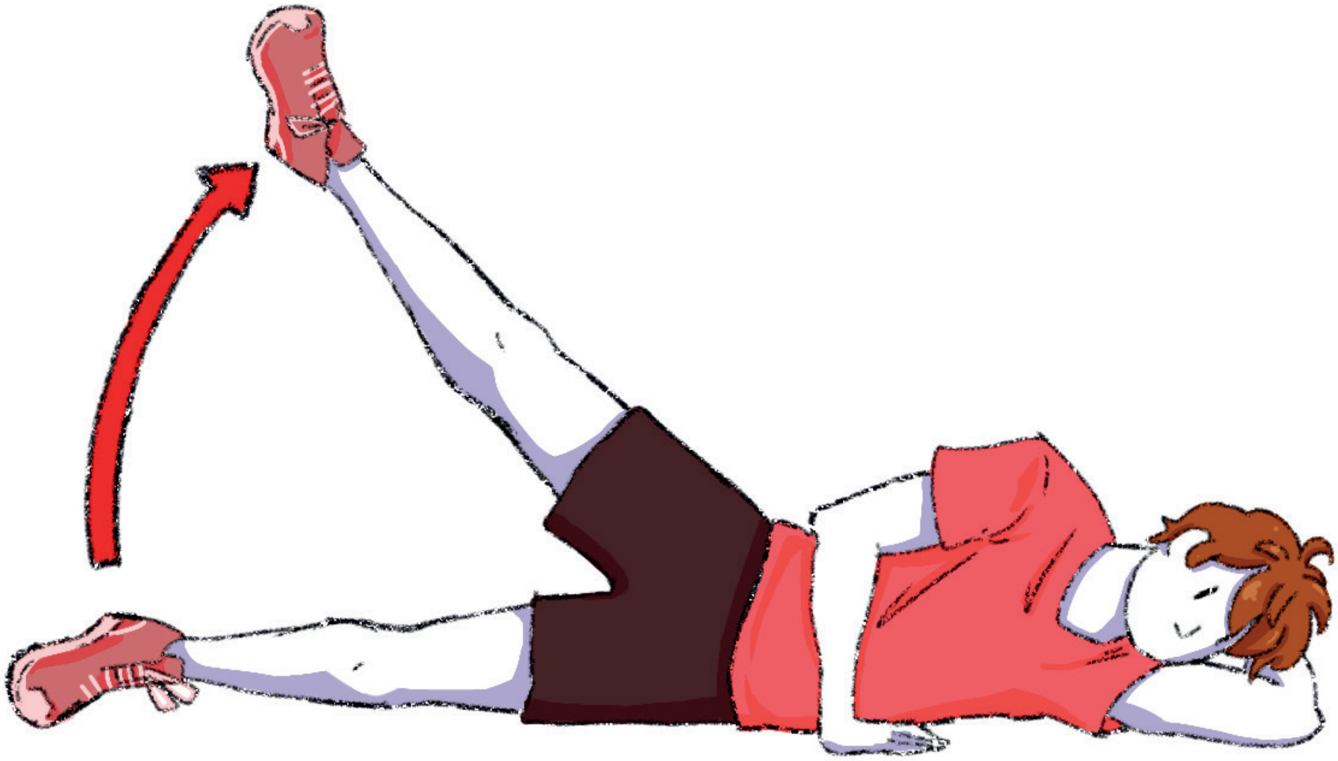

14. **RESPIRA E INSPIRA** – EXERCÍCIO RESPIRATÓRIO

Este exercício diminui a rigidez do tronco e está associado à melhora do sistema respiratório, pois exige uma grande quantidade de oxigênio a ser enviada para as células e músculos, tornando nosso organismo mais eficiente e beneficiando este sistema.

Em pé, com as mãos entrelaçadas atrás do pescoço, pés afastados na largura do quadril e coluna reta, puxe o ar pelo nariz enchendo completamente os pulmões. Com as duas mãos na nuca ainda, realize a rotação de tronco, soltando o ar pela boca. Retorne a posição inicial, puxando o ar pelo nariz enquanto se movimenta. Repita o movimento do outro lado, soltando o ar pela boca enquanto se movimenta. Repita por 10 vezes em 3 séries.

Séries: 3 **Repetições:** 10 **Intervalo de descanso:** 1 min

Exercício 14

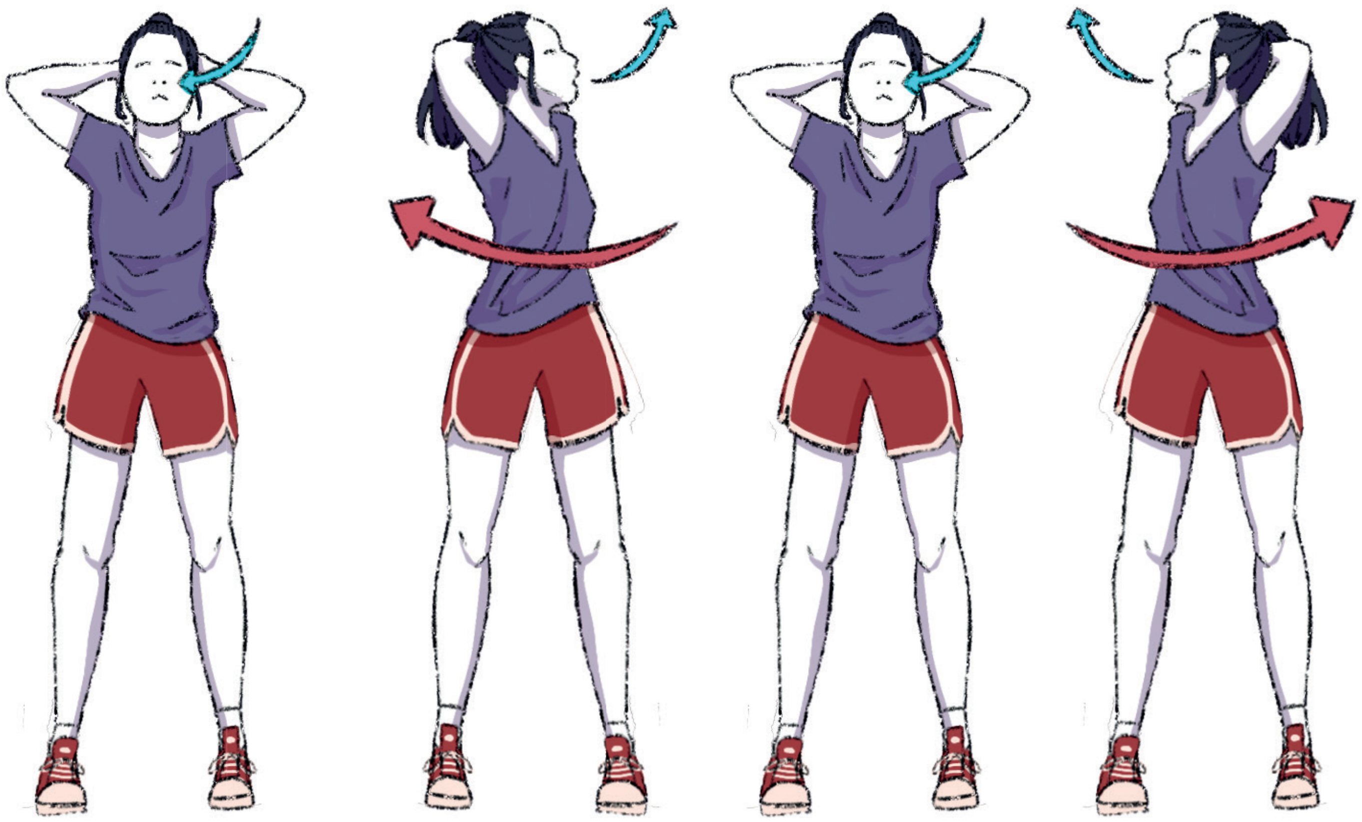

Exercícios para Prevenção de Quedas

Os exercícios a seguir tem como objetivo prevenir quedas por meio do fortalecimento muscular das pernas, treino de equilíbrio, coordenação motora e ganho de força e resistência.

15. SOBE E DESCE - EXERCÍCIO PARA EVITAR QUEDAS

Este exercício tem como finalidade treino do equilíbrio dinâmico e da coordenação motora e fortalecimento dos músculos das pernas para a caminhada, transferência e descarga de peso em pernas alternadas, e para subir e descer degraus de escada e calçadas.

Para a realização dessa atividade, é necessária uma escada ou calçada. Suba e desça um lance de escada durante um minuto. Descanse por 30 segundos e repita por 3 vezes o exercício.

Séries: 3 **Repetições:** 30 seg **Intervalo de descanso:** 1 min

Exercício 15

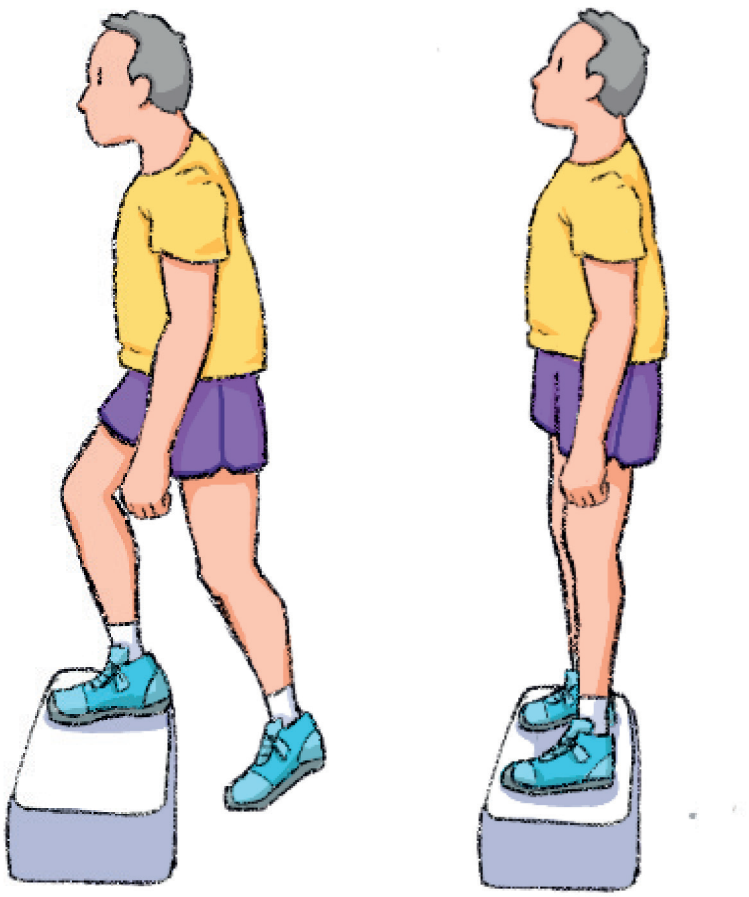

16. CAMINHADA EM CIRCUITO - EXERCÍCIO PARA EVITAR QUEDAS

Exercício 16

Este exercício em circuito tem como objetivo a melhora da força e da resistência muscular, da flexibilidade, da coordenação motora e do equilíbrio, contribuindo na prevenção de quedas e no desenvolvimento qualitativo de seu andar.

Realize uma caminhada em circuito em sua casa. Primeiro, realize o movimento com as pernas em linha reta e, em seguida, em um circuito com obstáculos, podendo ser caixas, cadeiras, tamboretes. Não esqueça de controlar a respiração soltando o ar entre os lábios. Repita por 3 a 5 minutos o circuito completo.

Atenção: Na execução deste circuito, retire qualquer objeto escorregadio do local, como: tapetes, esteira ou panos.

Séries: 3 **Repetições:** 3 a 5 min **Intervalo de descanso:** 1 min

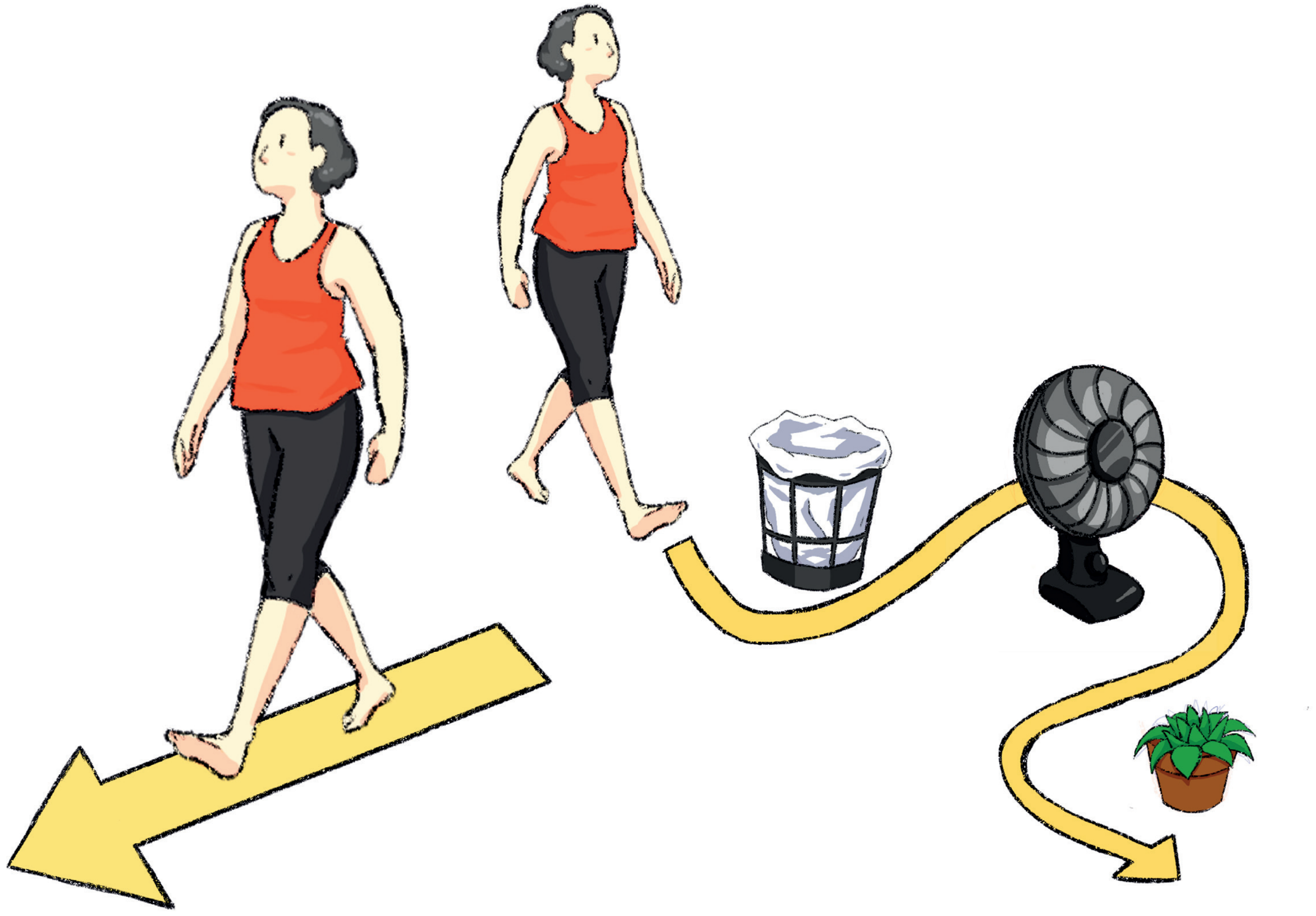

CURIOSIDADES

10 de novembro | Dia Mundial de Combate ao HTLV

O dia 10 de novembro, foi considerado pela Associação Internacional de Retrovirologia (IRVA – International Retrovirology Association), como Dia Mundial de Combate ao HTLV.

As ações de conscientização sobre a infecção pelo HTLV têm como objetivo difundir informações sobre o vírus para acabar com estigmas sobre a doença e erradicá-la mundialmente.

Representação existente no Brasil

HTLVida - Instituição criada para dar apoio ao portador do vírus HTLV localizada em Salvador-BA.

Site: http://www.htlvida.org/

E-mail: htlvida@bol.com.br

24 de novembro de 2021

Grande dia para saúde, para sociedade brasileira, e em especial, para as pessoas que vivem com HTLV. O Ministério da Saúde publicou a atualização do Guia de Manejo Clínico da Infecção Pelo HTLV. Com a implementação desse trabalho nas redes de saúde, espera-se que a assistência às pessoas que vivem com HTLV melhore e se dê uniformidade ao tratamento. Garantindo qualidade e dignidade a essas pessoas.

Para acessar o documento, clique aqui:

http://www.aids.gov.br/pt-br/pub/2021/guia-de-manejo-clinico-da-infeccao-pelo-htlv

AGRADECIMENTOS

Universidade Federal do Pará | Núcleo de Medicina Tropical

Programa de Pós-Graduação em Doenças Tropicais

https://portal.ufpa.br/

EBMSP - Escola Bahiana de Medicina e Saúde Pública

Grupo de Pesquisa Dinâmica do Sistema Neuro musculoesquelético da Escola Bahiana, Linha de Pesquisa HTLV

www.bahiana.edu.br

REFERÊNCIAS

ARAUJO, AQC, WEDEMANN, D. HTLV-1 Associated Neurological Complex. What is Hidden below the Water? AIDS Rev. 2019;21:211-217.

CASSEB. J.S.R. Manejo multidisciplinar de pessoas vivendo com HTLV, Ed. Lura Editorial, 2020.

CHAIYAWAT P,KULKANTRAKOM K. Randomized controlled trial of home rehabilitation for patients with ischemic stroke: impact upon disability and elderly depression.Psychogeriatrics.2012;12(3):193-99.

CONROY SS, ZHAN M, CULPEPPER WJ, ROYAL W, WALLIN MT. Self-Directed Exercise In Multiple Sclerosis: Evaluation Of A Home Automated Tele-Management System. J Telemed Telecare. 2017; 1: 1-10.

LIVRAMENTO DF, DURAN LS, GALVÃO-CASTRO B, SÁ KN. Efeito de Exercícios de Pilates na Postura de Portadores de HAM/TSP Associado ao HTLV-1. Revista Pesquisa em Fisioterapia. 2012; 2(1): 13-23.

PAES, LORENA DA SILVA. Tudo em Cima!: Exercícios físicos e qualidade de vida com HIV / Lorena da Silva Paes, Juliana Pereira Borges. - Rio de Janeiro: ABIA, 2010.

RUBIO AO, CABRERA-MARTOS I, RODRÍGUEZ-TORRES J, FAJARDO-CONTRERAS W, DÍAZ-PELEGRINA A, VALENZA MC. Effects of a Home-Based Upper Limb Training Program in Patients With Multiple Sclerosis: A Randomized Controlled Trial. Arch Phys Med Rehabil. 2016; 97(12):2027-33.

SANTOS, BRUNO BOTELHO. HTLV: saiba mais sobre a infecção do vírus parente do HIV. ATUALIZADO EM 28 DE JUNHO DE 2018. Acesso em 03/02/2020. https://www.ativosaude.com/saude-sexual/virus-htlv/

SANTOS, G.M., TAVARES, G.M.S., GASPERI, G., BAU, G.R. Avaliação mecânica da resistência de faixas elásticas. Rev Bras Fisioter, São Carlos, v. 13, n. 6, p. 521-6, nov./dez. 2009.

TANAJURA, T. Aspectos Neurológicos da Infecção pelo HTLV-1 na Bahia: resultados de um estudo de coorte de 8 anos. Tese (Doutorado), Faculdade de Medicina Bahia, Universidade Federal da Bahia, 2013.

TURNER AP, HARTOONIAN N, SLOAN AP, BENICH M, KIVLAHAN DR, HUGHES C et al. J Consult Clin Psychol. 2016 Apr;84(4):297-309.

WORLD HEALTH ORGANIZATION. INTERNATIONAL Classification of Functioning, Disability and Health: ICF [text on the Internet]. Geneva: WHO [cited 2011 Feb 15]. Available from: http://www.who.int/classifications/icf/en/.

DIÁRIO DE EXERCÍCIOS

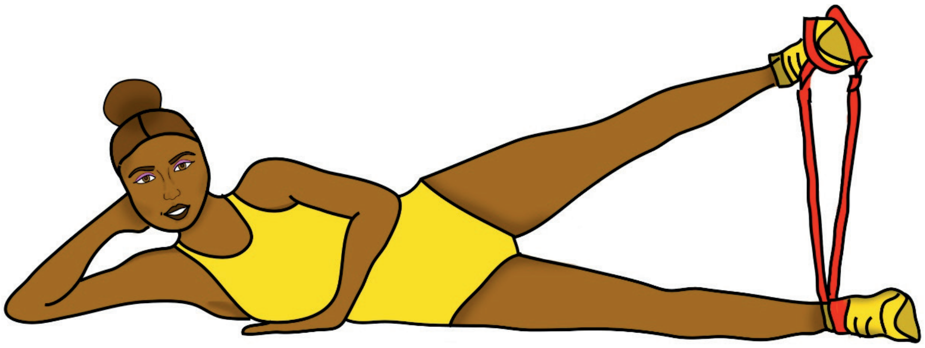

Desafie-se a fazer os exercícios três vezes na semana durante 05 meses. Faça o registro da atividade realizada, selecionando as opções: SIM, NÃO, UM POUCO.

**MÊS:**		
**DIA DA SEMANA**	**REALIZAÇÃO**	**ATIVIDADE REALIZADA (tipo de atividade realizada)**
DOMINGO	( ) Sim ( ) Não<br>( ) Um pouco	
SEGUNDA-FERIA	( ) Sim ( ) Não<br>( ) Um pouco	
TERÇA-FEIRA	( ) Sim ( ) Não<br>( ) Um pouco	
QUARTA-FERIA	( ) Sim ( ) Não<br>( ) Um pouco	
QUINTA-FEIRA	( ) Sim ( ) Não<br>( ) Um pouco	
SEXTA-FEIRA	( ) Sim ( ) Não<br>( ) Um pouco	
SÁBADO	( ) Sim ( ) Não<br>( ) Um pouco	

MÊS	Os exercícios permitiram que...<br>(benefícios da atividade física realizada)
1° MÊS	
2° MÊS	
3° MÊS	
4° MÊS	
5° MÊS	

ISBN: 978-65-00-68860-3
